# White matter damage as a consequence of vascular dysfunction in a spontaneous mouse model of chronic mild chronic hypoperfusion with eNOS deficiency

**DOI:** 10.1038/s41380-022-01701-9

**Published:** 2022-08-10

**Authors:** Xingyong Chen, Ling Chen, Geng Lin, Zhengjun Wang, Mahesh C. Kodali, Mingqi Li, Huimin Chen, Sarah G. Lebovitz, Tyler C. Ortyl, Lexiao Li, Saifudeen Ismael, Purnima Singh, Kafait U. Malik, Tauheed Ishrat, Fu-Ming Zhou, Wei Zheng, Francesca-Fang Liao

**Affiliations:** 1 Department of Pharmacology, Addiction Science, Toxicology, University of Tennessee Health Science Center, College of Medicine, Memphis, TN 38163, USA.; 2 Department of Neurology, Fujian Provincial Hospital, Shengli Clinical Medical College of Fujian Medical University, Fuzhou 350001, PR China.; 3 Department of Cell Biology and Genetics, The school of Basic Medical Sciences, Fujian Medical University, Fuzhou 350001, PR China.; 4 Teaching Center of Basic Medical Experiment, China Medical University, Shenyang, Liaoning 110122, PR China.; 5 Department of Anatomy and Neurobiology, University of Tennessee Health Science Center, College of Medicine, Memphis, TN 38163, USA.; 6 Department of Histology and Embryology, Basic Medical University, China Medical University, Shenyang, Liaoning 110122, PR China.; 7 These authors contributed equally: Xingyong Chen, Ling Chen, Geng Lin, Zhengjun Wang, Mahesh C. Kodali.

## Abstract

Vascular cognitive impairment and dementia (VCID) is the second most common form of dementia after Alzheimer’s disease (AD). Currently, the mechanistic insights into the evolution and progression of VCID remain elusive. White matter change represents an invariant feature. Compelling clinical neuroimaging and pathological evidence suggest a link between white matter changes and neurodegeneration. Our prior study detected hypoperfused lesions in mice with partial deficiency of endothelial nitric oxide (eNOS) at very young age, precisely matching to those hypoperfused areas identified in preclinical AD patients. White matter tracts are particularly susceptible to the vascular damage induced by chronic hypoperfusion. Using immunohistochemistry, we detected severe demyelination in the middle-aged eNOS-deficient mice. The demyelinated areas were confined to cortical and subcortical areas including the corpus callosum and hippocampus. The intensity of demyelination correlated with behavioral deficits of gait and associative recognition memory performances. By Evans blue angiography, we detected blood–brain barrier (BBB) leakage as another early pathological change affecting frontal and parietal cortex in eNOS-deficient mice. Sodium nitrate fortified drinking water provided to young and middle-aged eNOS-deficient mice completely prevented non-perfusion, BBB leakage, and white matter pathology, indicating that impaired endothelium-derived NO signaling may have caused these pathological events. Furthermore, genome-wide transcriptomic analysis revealed altered gene clusters most related to mitochondrial respiratory pathways selectively in the white matter of young eNOS-deficient mice. Using eNOS-deficient mice, we identified BBB breakdown and hypoperfusion as the two earliest pathological events, resulting from insufficient vascular NO signaling. We speculate that the compromised BBB and mild chronic hypoperfusion trigger vascular damage, along with oxidative stress and astrogliosis, accounting for the white matter pathological changes in the eNOS-deficient mouse model. We conclude that eNOS-deficient mice represent an ideal spontaneous evolving model for studying the earliest events leading to white matter changes, which will be instrumental to future therapeutic testing of drug candidates and for targeting novel/specific vascular mechanisms contributing to VCID and AD.

## INTRODUCTION

Vascular cognitive impairment and dementia (VCID), is the second most common form of dementia in the elderly after Alzheimer’s disease (AD) [1] and is caused by insufficient blood supply to the brain. Cerebrovascular pathology is present in at least half of all dementia cases [2–4]. VCID encompasses a broad spectrum of cerebrovascular-driven cognitive impairment, from mild cognitive impairment to fully developed dementia. This disease state is often further complicated by genetic factors (ApoE4), metabolic disorders (hypertension, type 2 diabetes, high cholesterol, hyperhomocysteinemia) and lifestyle factors (smoking, inactivity, excessive alcohol consumption) [5, 6]. These risk factors often result in chronic cerebral hypoperfusion and microvascular pathology [7–10]. They are the most common pathologies that co-occurs in VCID and AD [11, 12]. Although there are several good models to stimulate individual aspects of VCID [e.g., in vitro models of the neurovascular units, surgical models of chronic cerebral hypoperfusion induced by bilateral common carotid artery stenosis (BCAS), animals with NOTCH3 mutations as a model of small vessel disease], the heterogeneity of the disease states limits any of them from being a perfect model of all aspects of the disease [13]. Therefore, there is a pressing need to develop representative animal models to better understand the underlying mechanisms and identify preclinical biomarkers, prevention and treatment strategies.

Our prior work based on mice partially deficient in expressing endothelial nitric oxide (eNOS) gene has identified a potentially excellent model for studying the contribution of vascular dysfunction to dementia [14]. Endothelial eNOS-deficient mice spontaneously develop multiple hypoperfused/occluded areas, matching the most vulnerable areas of hypoperfusion in early AD patients [15–17]. Moreover, these mice also display microinfarctions, microbleeds, cerebral amyloid angiography, and hippocampus-dependent neurodegeneration in an age-dependent manner [14]. However, another invariant feature of VCID patients, namely, white matter pathology, was not studied previously.

White matter health is of paramount importance to normal cognitive performance [18]; the recent comprehensive transcriptomic study on AD forebrain using single-cell RNA sequencing on different cell types revealed that genes associated with the myelination pathways are the most changed group correlated with early stages of disease progression [19]. White matter tracts are a region at heightened risk for vascular damage [20]. The exact molecular and cellular mechanisms of white matter changes are unclear. Chronic hypoperfusion, blood–brain barrier (BBB) breakdown, oxidative stress, and inflammation have all been postulated as potential mechanisms [21–24]. In this study, we present data on all these aspects of cellular events concerning white matter changes in the eNOS-deficient mouse model.

## METHODS

### Materials and methods

#### Animals.

Mice deficient in eNOS gene expression (eNOS^−/−^, NOS3^tm1Unc^/J Jackson Laboratory, Bar Harbor ME) were crossbred with C57BL/6 mice to obtain eNOS^+/−^. Animals eNOS^+/−^, eNOS^−/−^ and littermate eNOS^+/+^ mice were derived from interbreeding between eNOS^+/−^ mice, and both sexes of mice were used in all experiments. Littermate mice were genotyped by genomic PCR using tail biopsy samples. For sodium nitrate (SN) feeding, NaNO_3_ (S5506, Sigma, St. Louis, MO, USA) was added to the drinking water at a concentration of 85 mg L^−1^ (1 mM) [25]. All mice were housed and aged in well-ventilated cages under standard laboratory conditions on a 12:12 h light–dark cycle with food and water ad libitum. All experimental animal procedures were conducted in accordance with the animal care standards of the National Institute of Health and were approved by the Institutional Animal Care and Use Committee of the University of Tennessee Health Science Center.

#### Immunohistochemistry.

Mice were perfused transcardially with ice-cold phosphate-buffered saline (PBS) followed by ice-cold 4 % paraformalde-hyde (PFA) solution in PBS. Mouse brains were then removed from the cranium. After post-fixation in 4% PFA in PBS at 4 °C overnight, brains were either processed for OCT-embedding after passing through 20% to a final of 30% sucrose solution prepared in PBS for 36–48 h at 4 °C until fully sank or transferred to PBS for vibratome sectioning of “fresh” tissue. Brains were then cut into serial coronal sections by Cryostat (CM1900; Leica, Heidelberger, Germany) (16 μm) or 30 μm thickness on a vibratome for immunostaining after permeabilization with 0.1% Triton X-100 (v/v) in 0.01 M PBS (pH 7.4) for 15 min, followed by blocking in 10% normal goat serum (NS02L; Sigma-Aldrich, Inc.) for 1 h at room temperature. Table 1 summarizes the primary antibodies used in immunohistochemistry. After incubation with primary antibodies diluted in blocking solution overnight at 4 °C, tissue sections were washed in PBS and incubated with the following secondary antibodies diluted in blocking solution for 60 min at room temperature: Alexa Fluor 594 or 488 conjugated goat anti-rabbit or mouse IgG (H + L) (Cat. A11037, A11032, A11034, A11029; 1:1,000; Thermo Fisher Scientific, Inc.). Sections were washed in PBS and counterstained with DAPI (Roche Diagnostics, Lot# 70237122, 1:1000). After washing with PBS three times, sections were mounted on microscopy slides and covered with Fluoromount-G (Cat. 00–4959-52; Thermo Fisher Scientific, Inc.). Fluorescence images were captured by an inverted fluorescence microscope (Olympus IX50, Olympus Optical, Inc.) or a Keyence fluorescence microscope (BZ-X800E, Keyence Corporation of America). Fluorescent signals were quantified by NIH’s Image J software.

#### Histochemistry.

Luxol Fast Blue (LFB) staining was performed following the manufacturer’s protocol (NovaUltra Luxol Fast Blue Stain Kit, Cat IW-3005, IHC World). To measure brain reactive oxygen species (ROS) production, the brain sections were exposed to dihydroethidium (DHE) (D1168; Thermo Fisher Scientific, Inc.), according to a previously published validated method. Briefly, brain sections were incubated in PBS for 30 min at 37 °C and then encircled with the hydrophobic pen. DHE (5 mmol/L) was topically applied, followed by incubation at 37 °C in the dark for 30 min. Sections were rinsed in PBS, and oxidized DHE fluorescence was detected with an Olympus inverted fluorescence microscopy, and the fluorescence staining was quantified by ImageJ. Hypoxyprobe-1 staining. Mice were injected i. p. with 60 mg/kg pimonidazole hydrochloride (Hypoxyprobe^TM−1^ Plus Kit, HP2–100Kit, Natural Pharmacia International). Sixty minutes following injection, mice were sacrificed, and brains were dissected and fixed as specified for routine histological analysis. The formation of pimonidazole adducts was detected by immunostaining with an Hypoxyprobe-1-Mab1 FITC antibody and developed by DAB (Abcam) according to the manufacturer’s instructions.

#### Cerebral fluorescent angiography.

Cerebral fluorescein isothiocyanate (FITC)-dextran fluorescent angiography was performed as described in detail previously [14] using FITC-dextran (FD2000S-1G; 2000 kDa; 0.1 mL 50 mg/mL in sterile saline; Sigma, St. Louis, MO, USA). Mice were euthanized without perfusion 5 min after injecting FITC-dextran via tail vein and whole brains removed and post-fixed in 4% PFA in PBS at 4 °C overnight. Sequential coronal sections (100 μm thickness) were processed using vibratome for fluorescent imaging.

#### Evans blue angiography.

Evans Blue (E2129, Sigma, St. Louis, MO, USA) was injected in 150 μL of 2% (m/v) in sterile saline through mouse tail vein. Mice were euthanized 5 min later without perfusion and whole brains removed and post-fixed in 4 % PFA in PBS at 4 °C overnight. Sequential coronal sections (100 μm thickness) were processed using vibratome for fluorescent imaging using rhodamine filter of inverted fluorescence microscopy (Olympus IX50, Olympus Optical, Inc.). Serial images were taken at Bregma: 2.0, 1.42, 0.26, −0.22, and −1.82 mm. For cerebral double fluorescent angiography, mice received a mixture of FITC-dextran and Evans blue dye in 150 μL volume and euthanized 5 min later without perfusion. Brain tissue was processed for individual angiography, and some sections were also immune-stained with selective antibodies (e.g., BMP4 and MMP9).

#### White matter micro punch.

Mice were euthanized by isoflurane overdose and brains were excised and placed in ice-cold PBS. Brains were subsequently sectioned with a vibratome to obtain two 1 mm coronal sections containing the genu and body of the corpus callosum. White matter microsamples were collected via micro punch using a 0.5 mm blunt-end needle attached to an air-filled syringe under a dissecting microscope. Micro punches of the corpus callosum were collected and aspirated into microcentrifuge tubes in 0.5 mL TRIzol (Invitrogen) and stored at −80 °C until RNA extraction. The corresponding size of gray matter tissue was collected via micro punch from the adjacent subcortical region as control.

#### Bulk RNAseq of the micro punched samples.

Total RNA was extracted from micro punched samples using the RNeasy Mini kit (Qiagen, 74104) and submitted to Novogene for RNA-Seq library construction and sequencing. Briefly, messenger RNA was purified from total RNA using poly-T oligo-attached magnetic beads. After fragmentation, the first strand cDNA was synthesized using random hexamer primers followed by the second strand cDNA synthesis. The library was ready after end repair, A-tailing, adapter ligation, size selection, amplification, and purification. The library was checked with Qubit and real-time PCR for quantification and bioanalyzer for size distribution detection. Quantified libraries will be pooled and sequenced on Illumina platforms, according to effective library concentration and data amount. Transcript abundance from RNA-seq reads were quantified using Salmon [26], and gene-level counts were obtained using tximport [27], against the C57BL/6J mouse genome annotation Genome Reference Consortium Mouse Build 39 (GRCm39), obtained from the National Center for Biotechnology Information. Raw counts were processed with DESeq2. All statistical analyses were conducted using R version 4.0 as described in our recent publication [28].

#### Western blot analysis and quantitative real-time RT-PCR (qRT-PCR).

Western blots and qPCR were performed as described previously [14]. Briefly, mice were transcardially perfused with ice-cold PBS and brains were removed. Half brains were post-fixed and used in immunohistochemistry and the other half brains were further dissected to forebrain tissue and then homogenized in cell lysis buffer (20 mM Tris-HCl (pH 7.5), 150 mM NaCl, 1 mM Na_2_EDTA, 1% NP-40, 1% sodium deoxycholate 0.1% SDS) with complete protease inhibitor cocktail (Roche, Inc.). Lysate preparation and Western blot analysis were performed as previously described [16]. For qPCR, RNA was extracted from the dissected brain tissue using TRIzol according to the manufacturer’s instructions. Single-stranded cDNA was synthesized from 1 μg of total RNA using High Capacity cDNA Reverse Transcription kits (Applied Biosystems). qPCR was performed with RealM-asterMix SYBR Green (Applied Biosystems). Primary antibodies and qPCR primers used are summarized in Tables 1 and 2, respectively.

#### Brain slice electrophysiology for recording evoked extracellular compound axon action potentials (CAPs).

Eighteen (18) month-old eNOS^+/+^, eNOS^+/−^, eNOS^−/−^ and sodium nitrate (SN)-treated eNOS^−/−^ (SN eNOS^−/−^, 5-month treatment) mice were used to produce brain slices that contained the anterior portion of the brain including the corpus callosum. SN treatment via drinking water was described above. Under deep ketamine and xylazine anesthesia, mice were intracardially perfused with 30 mL oxygenated ice-cold cutting solution (detailed below) to cool and protect the brain and improve brain slice viability; then the brain was dissected out quickly and immediately immersed in the oxygenated ice-cold cutting solution (in mM: 220 glycerol, 2.5 KCl, 1.25 NaH_2_PO4, 25 NaHCO_3_, 0.5 CaCl_2_, 7 MgCl_2_, 20 d-glucose) for 2 min. Four hundred (400)-micrometer-thick coronal brain slices (3 slices per mouse, 3–4 mice/group) were cut on a Leica Zero Z VT1200S vibratome (Leica Microsystems, Wetzlar, Germany). The slices were transferred to a standard extracellular bathing solution (in mM: 125 NaCl, 2.5 KCl, 25 NaHCO_3_, 1.25 NaH_2_PO_4_, 2.5 CaCl_2_, 1.3 MgCl_2_, and 10 d-glucose) that was continuously bubbled with 95% O_2_-5% CO_2_ for 30 min at 34 °C in a holding chamber, and then the holding chamber was kept at room temperature (20 °C). Brain slices were used within 1–5 h after being prepared. Ascorbic acid (vitamin C, 0.4 mM) was added to all solutions to protect the brain tissue and improve slice viability.

For recording evoked extracellular compound axon action potentials (CAPs), a brain slice was placed in a recording chamber and continuously perfused at 2 mL/min with the standard extracellular bathing solution (detailed above) saturated with 95% O_2_–5% CO_2_. Recordings were made under visual guidance of a video microscope (Olympus BX51WI and Zeiss Axiocam MRm digital camera) with a video monitor that was calibrated with a stage micrometer (graticule) that can measure distance with a 10 μm resolution. A Multiclamp 700B amplifier, pClamp 9.2 software, and Digidata 1322A interface (Molecular Devices, Sunnyvale, CA) were used to acquire data. Patch pipettes were pulled from borosilicate glass capillary tubing (BF150-110-10, 1.1-mm ID, 1.5-mm OD; Sutter instrument, Novato, CA) with a PC-10 puller (Narishige, Tokyo, Japan), had resistances of 2 MΩ and filled with the standard extracellular bathing solution for recording CAPs. Electrical signals were filtered at 10 kHz with the built-in four-pole low-pass Bessel filter in the patch-clamp amplifier and digitized at 50 kHz (20 μs/sample). All recordings were made at 20 °C such that the CAP conduction was relatively slow and CAPs were separate from the stimulus artifact. At least four sweeps were collected from each brain slice to obtain averaged CAPs. Conventional electrical stimulation was used to evoke CAPs. A bipolar tungsten stimulating electrode (World Precision Instruments, Sarasota, FL) was placed superficially into corpus callosum (CC). Stimulating pulses were controlled by a pulse generator (Master-8; AMPI, Jerusalem, Israel) and delivered via a stimulus isolator (A365; World Precision Instruments, Sarasota, FL). A single stimulating pulse was delivered every 20 s to evoke CAPs. A relatively low stimulation intensity (ranged from 200 to 500 μA) with a constant duration of 0.2 ms. CAP data were analyzed using Clampfit 9.2 software (Molecular Devices, Sunnyvale, CA) and presented as mean ± standard error. Two-way ANOVA and posthoc Tukey’s test were used.

#### Gait balance test (CatWalk).

Mice were subjected to gait assessment using the CatWalk (Noldus Information Technology, Wageningen, The Netherlands) which consists of an enclosed walkway on a glass plate that is traversed by mice from one side of the walkway to the other. The room light is off. A camera is set below the glass floor to record paw prints. The red light installed inside the deck generates the background in the video. The glass floor sheds green fluorescence-like light so that paw prints are distinguished when the paw touches the floor. The digitalized paw prints are then automatically classified into four cohorts, namely left forepaw, left hind paw, right forepaw, and left hind paw. Mice from the same housing cage were trained to traverse a glass walkway toward their home cages from one end to another without interruptions for 30 min for five consecutive days. On subsequent test day, three non-stop complete runs across the walkway were recorded. The test starts once the mouse enters the visual field of the camera and stops once the mouse disappears. If an animal failed to complete a run, walked backwards, or reared during the run, it was given an additional run. Quantitative analysis of gait performance was done by CatWalk XT version 10.0.408 to include the (1) spatial parameters related to individual paws (intensity, maximum area, print area, etc); (2) relative spatial relationship between different paws (base of support, relative paw placement, and stride length); (3) interlimb coordination (step pattern, regularity index, and phase lag); and (4) temporal parameters (swing, stance, cadence, and walk speed). Data are presented as mean ± s.e.m.

#### Novel objective recognition (NOR) test.

This test, based on the spontaneous tendency of animals to explore and interact with a novel object more than a familiar one, typically consists of 2 trials separated by a retention period and preceded by a habituation phase. The habituation phase (15 min/d) will be conducted on 2 separate days, before the start of the test, to allow animals to acclimate to their arena, which consisted of an empty standard size box (45 × 45 × 35 cm). On the designated test day, each animal will be first introduced into the box containing 2 identical sample objects (acquisition/sample trial) and will be allowed to explore these for 10 min (19). Following sample object exposure, the animal will be returned to its home cage for a 1 h retention period. The 2nd preference/test trial (15 min), which follows the retention period, will be conducted in the same manner as the 1st trial, except that a new/novel object replaced one of the familiar/sample objects. The arena and objects will be cleaned after each session with 70% ethanol. All trials will be recorded, and video tracked by ANY-Maze automated tracking system. The criteria for “object exploration” will be met, when the animal’s head is oriented toward the object and the animal either touches the object with its nose or sniffs at it, such that the physical nose-object distance is ≤2 cm. Data analysis includes calculation of the discrimination index and recognition index and performing one-way ANOVA to determine *p* values.

#### In vitro BBB models.

Murine bEnd.3 cell line (ATCC, CRL-229) was isolated from brain tissue derived from BALB/c mouse with endothelioma and was is sub-cultured at a 1:3 to 1:4 split ratio approximately every 3 days. These cells were widely used in both monolayer and in co-culture with astrocytes and pericytes in transwells to establish BBB models and were both established as valid in vitro models based on the barrier integrity as determined by the transendothelial electrical resistance/TEER [29]. We have compared the bEnd.3 monolayer with the eEnd.3 and C8-D1A (astrocyte type I clone, ATCC, CRL-2541) co-culture (with either bEnd3 on top or on bottom in transwells) and repeated the experiments in both systems (*N* = 3–4 on each assay of Western blots and TEER measurement). Cells were treated with murine recombinant rBMP4 (R&D 5020-BP; 25 ng/mL) for various time points with or without adding BMP4 inhibitor (LDN 193189 dihydrochloride, 100 nM, Bio-Techne Corporation). TEER was determined using the device model (ECIS 1600R, Applied Biophysics, Troy, NY).

#### Statistical analysis.

Statistical analyses were performed using unpaired two-tailed or one-tailed Student’s *t*-test. For western blot quantification, statistics were performed using one-way ANOVA test. Data are reported as mean ± s.e.m. (stand error of the mean), except as mean ± s.d.m. (standard deviation of the mean) for qPCR. Statistical significance was set at *P* < 0.05.

## RESULTS

### Middle-aged eNOS-deficient mice display severe myelin loss and axonal pathology

**Fig. 1:**
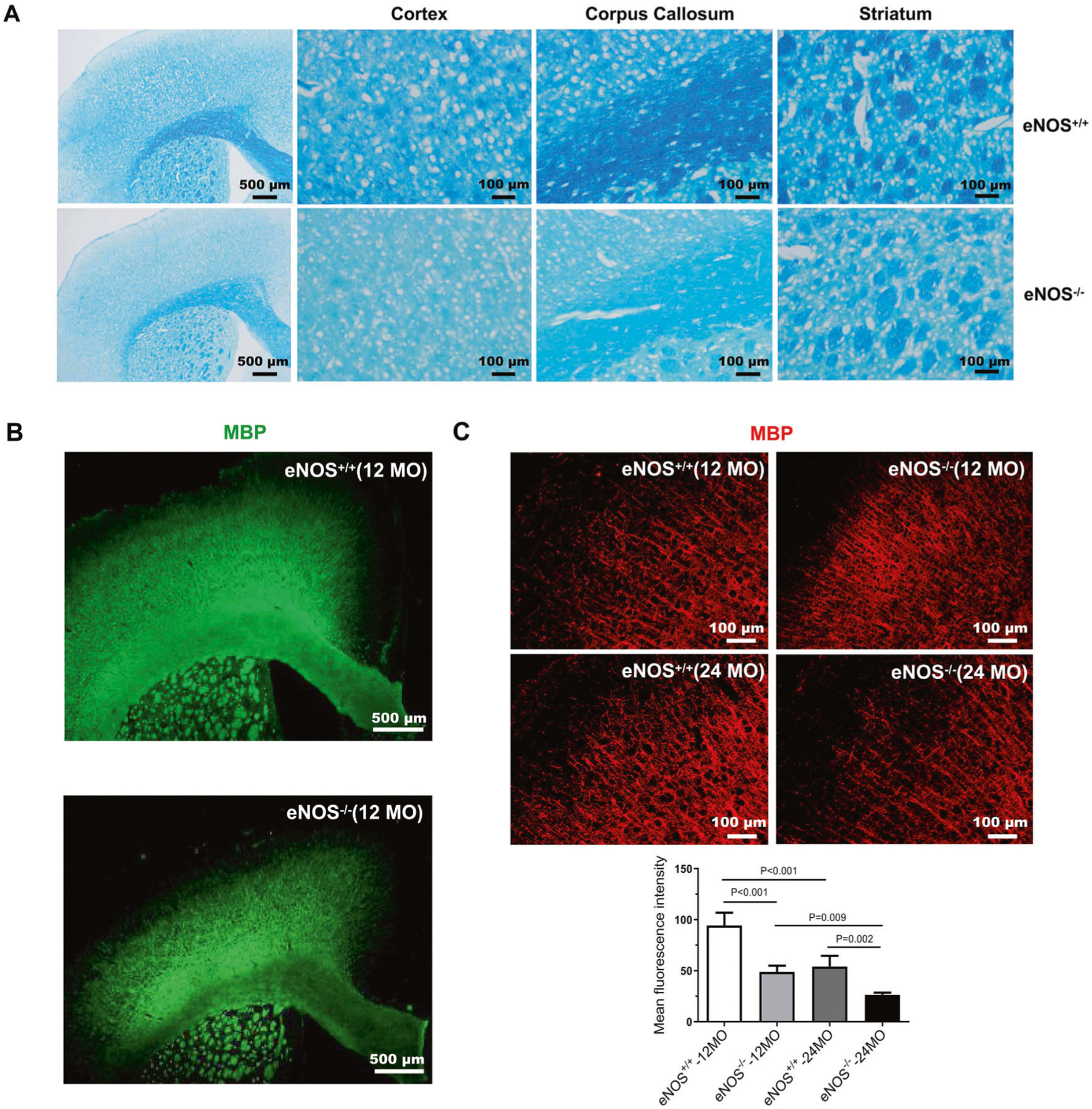
Severe myelin loss in middle-aged eNOS-deficient mice. **A** Representative images of LFB (coronal, 16 μm) staining taken from mice at 12 months of age. Scaled bars are indicated in each picture. **B** Representative images of MBP immunohistochemistry at 12 months of age (12 MO). Scaled bars: 500 μm. **C** Representative images of MBP immunohistochemistry from the frontal cortical regions at 12 months of age. Scaled bars: 100 μm. Quantification was performed based on the mean fluorescent intensity from the frontal cortical regions and expressed as mean ± s.e.m; *N* = 5–6 mice/group.

In our previous work, we characterized AD-like pathologies in eNOS-deficient mice at old age (>15 MO) [14]. We have recently focused on characterizing middle-aged mice (12 MO), since metabolic vascular factors pose the highest risk in this age group [5, 6]. As reported, eNOS-deficient mice manifested most of the phenotypes of metabolic syndrome such as hypertension, insulin resistance, hyperlipidemia, hypertension, and hyperhomocysteinemia [30–32]. We now determined white matter changes at three ages (7 MO, 12 MO, and 24 MO) in eNOS-deficient (eNOS^−/−^) and littermate control mice (eNOS^+/+)^. Coronal sections encompassing the brain regions corresponding from frontal to temporal cortical areas [+2.0 mm to −2.5 mm] were systematically examined by histological means for the expressional changes in axonal and myelin protein markers. Firstly, myelin integrity was assessed in eNOS mouse brains by both LFB (luxol fast blue) staining (Fig. 1A) and immunohistochemistry on myelin basic protein (MBP) (Fig. 1B). Significant myelin loss was detected in the cortex and corpus callosum (CC), but not in the striatum in eNOS^−/−^ mice, starting from 7 months of age; quantified data at 12 and 24 months of age are presented (Fig. 1 and SFig. 1). Myelin loss was also detected in eNOS heterozygous mice at mid-age (SFig. 1B) and thinning of the CC region was detected at old age (24 MO).

Surprisingly, forebrain western blot data showed a nearly twofold increase in MBP protein in eNOS^−/−^ at mid-age on all four alternatively spliced isoforms, predominantly with 17 kDa and 21.5 kDa species (SFig. 1C) which presumably play differential roles in oligodendrocyte functions. Notably, there was markedly increased colocalization of MBP immunosignals in the nuclei of eNOS^−/−^ brains (SFig. 1D), which may represent mis-aggregated dysfunctional protein. Nuclear MBP reportedly displays different roles in myelination compared to the cytoplasmic protein species, most often seen with the 17 and 21.5 kDa isoforms [33], appearing to be associated with the process during oligodendrocyte maturation before compact myelin formation [34]. In addition, immunoreactive signals of two other myelin-pathway protein markers MOG (myelin oligodendrocyte glycoprotein) and PLP (myelin proteolipid protein) were reduced in selective brain regions in eNOS-deficient brains (SFig. 2A–C). Accordingly, all four major myelin-pathway genes (i.e., *Mbp, Plp1*, *Mog*, and *Mag*) were found to be markedly downregulated in the CC region in young eNOS^−/−^ mice (SFig. 2D).

### Impaired associative recognition memory, gait imbalance and cortical pyramidal neurodegeneration were detected in middle-aged eNOS-deficient mice

**Fig. 2:**
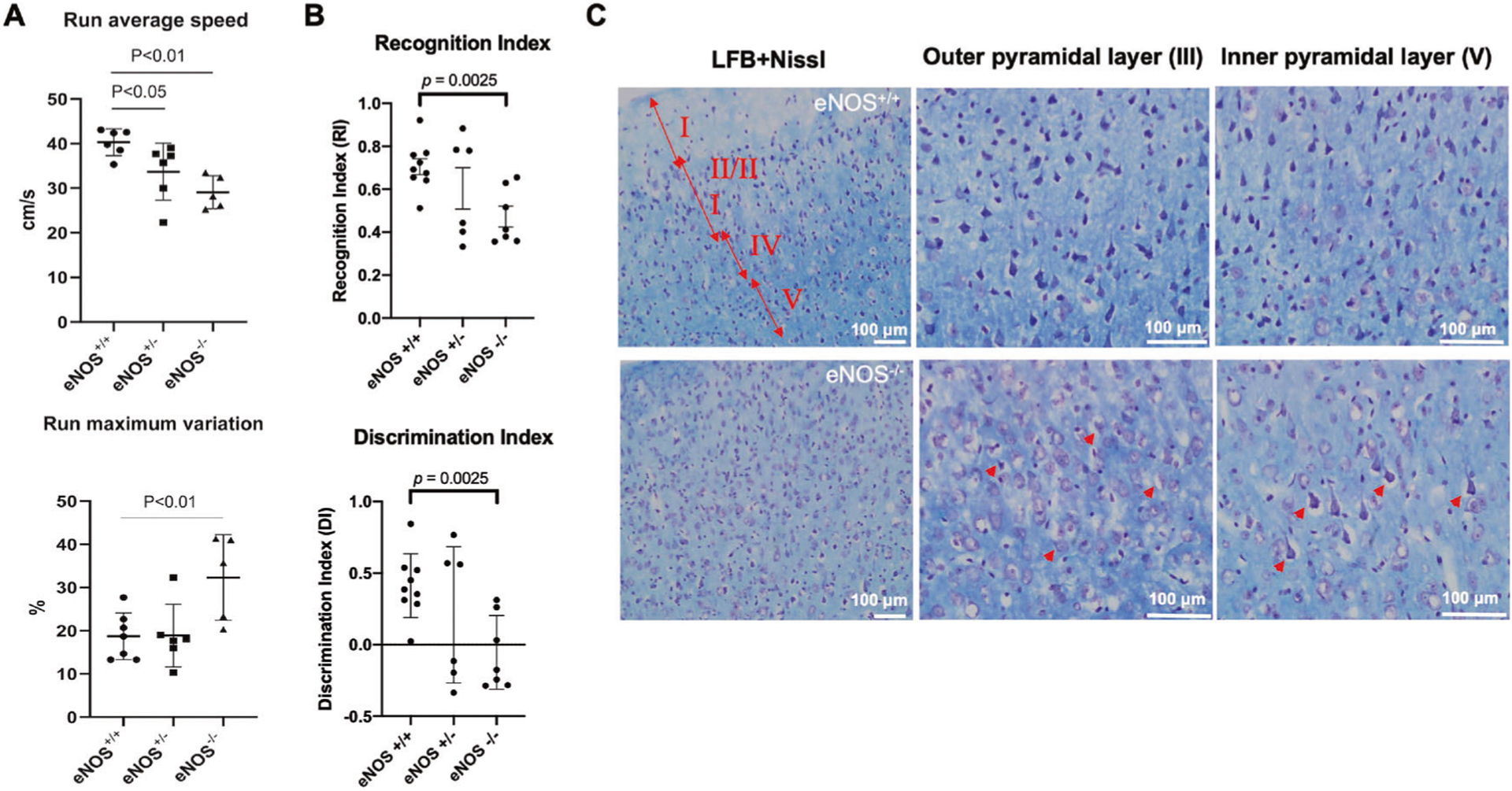
Gait disbalance, impaired associated recognition memory and cortical pyramidal neurodegeneration at early mid-age. **A** Gait performance by CatWalk testing. The test started once the mouse entered the visual field of the camera, and stopped once the mouse disappeared. The averaged running speed and additional parameters (e.g., altered sequence patterns and interlimb coordination) were recorded, analyzed, and expressed as mean ± s.e.m; *N* = 5–7 mice/group per genotype (11–12 MO). **B** The novel objective recognition (NOR) test was used to assess associated recognition memory in eNOS^+/+^, eNOS^+/−^, and eNOS^−/−^ mice (9 MO). The eNOS^−/−^ mice showed a significant reduction in discrimination index (DI) and recognition index (RI), evident by their inability to discriminate and recognize between the novel and familiar objects, compared to littermate eNOS^+/+^ mice (mean ± s.e.m; *N* = 6–9). **C** Representative images of LFB and Nissl double-stained sections of brains of eNOS^−/−^ and control littermates showing frontal cortical regions at 12 months of age. Red double arrows and the corresponding Roman numerals indicate cortical layers I through V. Red arrowheads indicate degenerating neuron with swollen soma. Scaled bars are indicated in each picture.

Cortical white matter change is often associated with gait dysfunction in humans. We used the CatWalk system to assess gait performance of eNOS littermate mice in all three genotypes at 12 months of age. Figure 2A shows that the averaged running speed was consistently reduced in eNOS^+/−^ mice and was even slower in eNOS^−/−^ mice. Of note, eNOS^−/−^ mice not only moved slowly, but also failed to run at a constant speed compared to eNOS^+/+^ and eNOS^+/−^ mice. Moreover, eNOS-deficient mice (both eNOS^+/−^ and eNOS^−/−^) displayed significantly altered patterns of gait sequence interlimb coordination compared to eNOS^+/+^ mice. The same cohort of eNOS-deficient mice displayed significant deficits in two additional motor tests of rotarod and grip strength (data not shown).

Novel objective recognition (NOR) test is commonly used to measure associative recognition memory dependent on an intact cortical neuronal circuit [35]. We found that the NOR performance was severely impaired in young eNOS^−/−^ mice (Fig. 2B), followed by hippocampus-dependent spatial working memory deficits at older age (15–18 MO) as we reported earlier [14]. Based on clinical evidence, the executive function in this associated cortical task is generally defective in individuals with mild cognitive difficulty, prior to the progression to hippocampus-dependent long-term learning and memory in severely demented patients [36].

Cortical white matter demyelination predictably leads to cortical circuit dysfunction. We thus examined the overall health of the neurons in both cortical and hippocampal regions of eNOS^−/−^ and control mice by LFB-Nissl double staining. We detected disorganized cortical structure with signs of severe neurodegeneration of cortical pyramidal neurons in layers II/III and V/VI at 12 months of age, revealing the presence of an empty vacuole surrounding the seemingly degenerated neurons (Fig. 2C), presumably due to loss of myelin sheath; no apparent neurodegeneration of hippocampal neurons was detected at this age.

### Elevated ROS and astrogliosis and neuroinflammation detected selectively in the white matter before the middle age

**Fig. 3:**
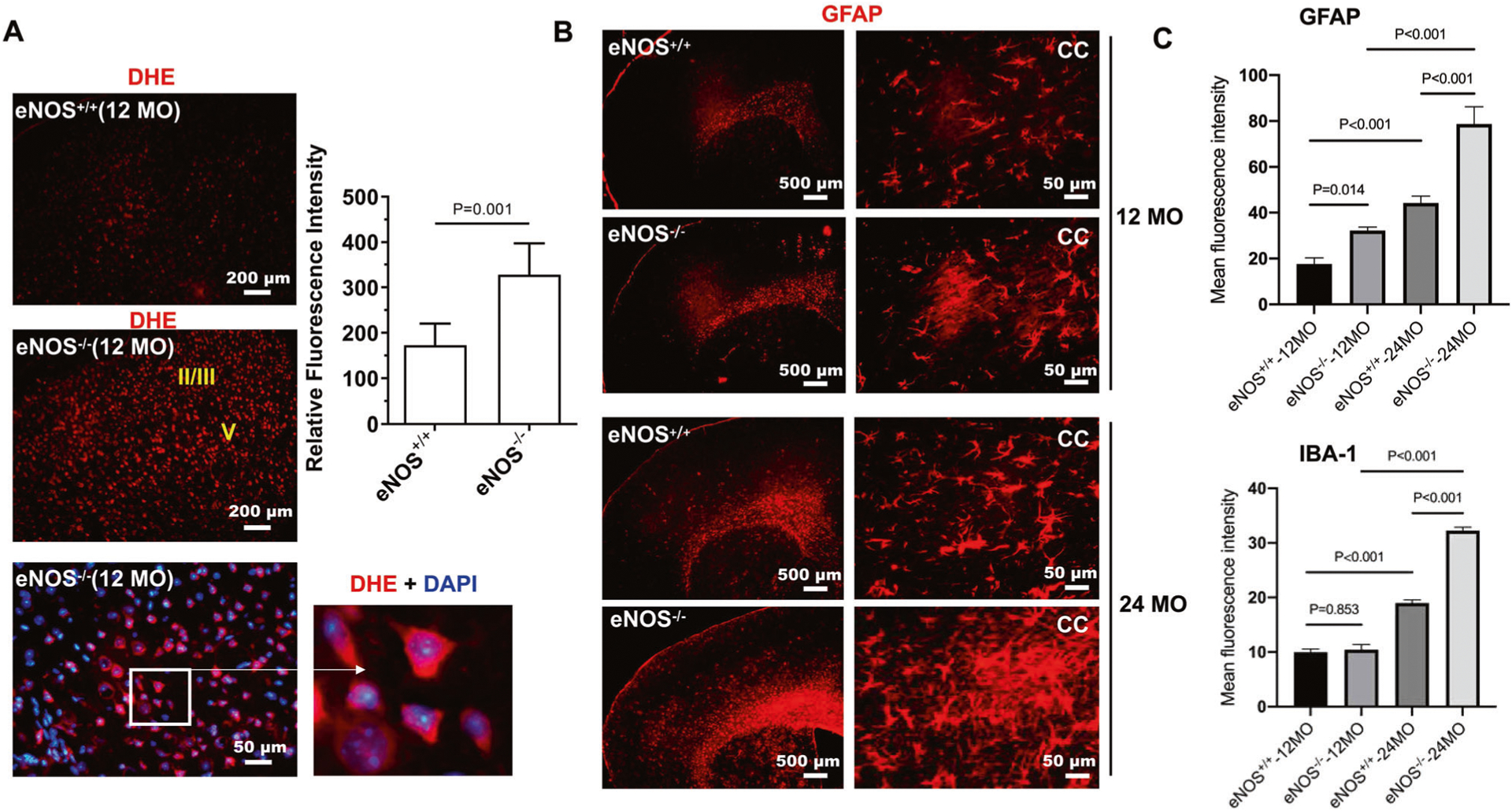
Elevated reactive oxygen species (ROS) and astrogliosis. **A** Representative images of dihydroethidium (DHE)-stained frontal forebrain cortical regions from mice at 12 months of age. Roman numerals in yellow indicate cortical layers II/III and V where we detected the most elevated ROS signals. Lower right panel images with zoned the area showing intracellular DHE signals presumably deriving from the clustered mitochondria in the cytoplasm. Quantification was performed based on the mean fluorescent intensity from the frontal cortical regions and expressed as mean ± s.e.m; *N* = 5–6 mice/group. **B** Representative images of GFAP immunohistochemistry on coronal brain sections of mice at 12 and 24 months of age. **C** Quantification of GFAP and Iba-1 immunofluorescence intensity based on 5–6 mice/group and expressed as mean ± s.e.m.

We observed markedly elevated ROS levels by DHE staining, selectively in the parietal cortical region (Fig. 3A, SFig. 3A). Furthermore, we found markedly increased fibrous astrocytes based on the expression of the glial fibrillary acidic protein (GFAP) in the CC region in middle-aged eNOS-deficient mice; they were aligned in rows between the axon bundles in parallel, which displayed smooth and long processes of a fingerlike shape (Fig. 3B). Heterozygous eNOS^+/−^ brains also displayed an increase in GFAP-positive cells at mid-age, but at lesser degrees than eNOS^−/−^ brains (SFig. 3B). Surprisingly, there was no significant increase in Iba-1 positive microglia in a mid-age; a significant global increase was found at 24 months of age (Fig. 3C).

Although GFAP is the main astrocytic intermediate filament, immunohistochemistry using multiple GFAP antibodies only labeled astrocytes in the corpus callosum, cerebral peduncle, and hippocampus in mouse brain. Owning to the increasingly recognized heterogeneity of astrocytes from different brain regions [37], we used a second marker S100β which is more suitable for capturing a global brain distribution of astrocytes. Indeed, we observed more S100β-positive astrocytes in the cortical area in eNOS^−/−^ brain at mid-age; not only the number but also the fluorescent intensity of each astrocyte was markedly increased (SFig. 3C). Contrary to the globally increased S100β-positive astrocytes in both the CC and cortical areas in eNOS^−/−^ brains, the GFAP-positive astrocytes were selectively found around the cortical layer II/III and V/VI regions, surrounding the degenerating/degenerated pyramidal neurons (SFig. 3D). Astrocytes are best known for multi-faces (e.g., tissue repair, and mediating an inflammatory response). Indeed, at higher magnification, both the GFAP- and S100β-positive astrocytes displayed a thicker cellular process, indicating reactive astrocytes upon injury (SFig. 3C and 3D). Accordingly, we also detected markedly upregulated levels of three pro-inflammatory cytokine genes only in the white matter of the eNOS^−/−^ brain at 10 months of age (SFig. 3E), indicating white matter neuroinflammation may be an early contributing mechanism to the cortical pathology such as demyelination and selective cortical neurodegeneration.

### Mild regional hypoperfusion and BBB leakage are the two earliest pathologies detected in eNOS-deficient mice

**Fig. 4:**
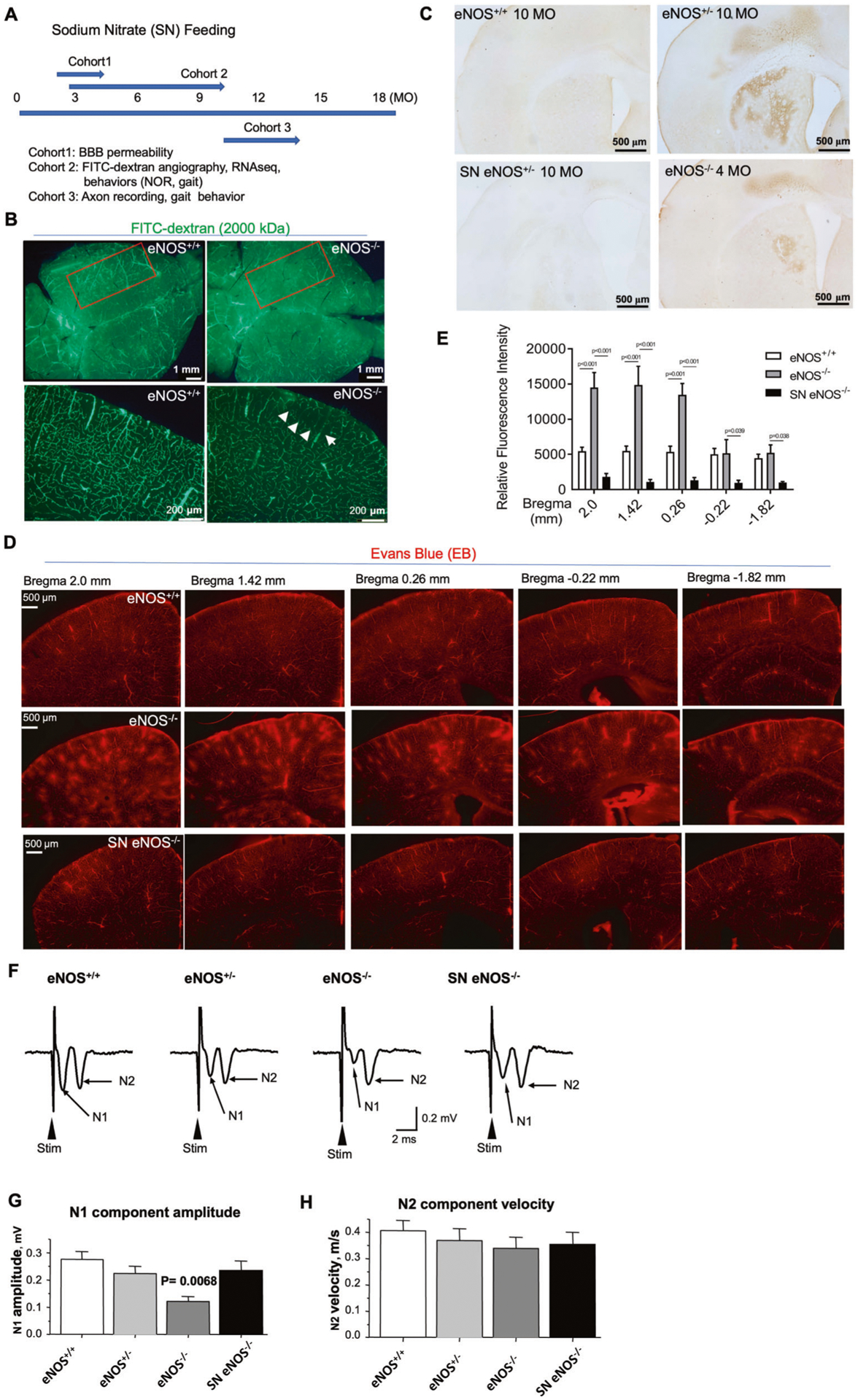
Functional recovery after sodium nitrate feeding. **A** Schematic diagram illustrating sodium nitrate (SN) feeding schedules in three cohorts of mice. **B** Representative FITC-dextran angiograms of whole brain (left two panels) from dorsal view from eNOS mice at 3 months of age. Red boxed indicates the parietal cortical zone from the dorsal view. The right two panels show the representative FITC angiograms taken from 100 mm coronal sections of frontal brains from the same mice as in the left two panels. **C** Representative images of hydroxyprobe images. **D** Representative images of Evans blue angiograms of young eNOS^+/+^ and eNOS^−/−^ mice (3 months of age) taken at 2.0 to −1.82 mm Bregman, showing BBB leakage detected at a young age which can be prevented by sodium nitrate (SN) feeding in drinking water for 6 weeks starting from 8 weeks old (SN eNOS^−/−^, *N* = 3 mice/groups). Evans Blue (EB, 2 % in sterile water) was injected in 150 μL volume through mouse tail vein. Mice were euthanized 5 min later without perfusion and whole brains removed and vibratome-processed to 100 μm serial sections for fluorescent imaging using an rhodamine filter. **E** Quantification of Evans blue fluorescent signals based on the angiograms presented in panel. Data are presented as mean ± s.e.m based on *N* = 3 mice each group. **F**–**H** Examples of compound axon action potentials (CAPs) recorded in the corpus callosum in eNOS^+/+^ (WT) mice, eNOS^+/−^ mice, eNOS^−/−^ mice and sodium nitrite-treated (SN) eNOS^−/−^ mice. **G** Summary of the amplitudes of the myelinated axon-generated fast N1 component in the four groups of mice. **H** Summary of the amplitudes of the unmyelinated axon-generated slow N2 component in the four groups of mice.

Endothelium-derived NO (EDNO) signaling has long been known to be crucial for angiogenesis and vasculogenesis during the early developmental stage. Using FITC angiography, we detected reduced fluorescent signals from a dorsal view of eNOS-deficient mice at a young age (3 MO) (Fig. 4B, left panels). We reasoned that this phenomenon indicated arterial occlusion resulting from impaired NO signaling. Indeed, as we reported previously [14], regions of the hypoperfusion and microvascular occlusion as evidenced by the lack of FITC-dextran distribution appeared in eNOS-deficient mice at a young age, with markedly increased frequency of similar lesions in an age-dependent manner. As shown in the right panels of Fig. 4B (white arrows), these lesions/occlusions are typically small (100–200 μm in diameter); by middle age, they were frequently found bilaterally in the parietal association cortex (Table 3). This pathological pattern is highly reminiscent of the bilateral temporoparietal hypoperfusion characteristic of AD patients [14]. Using hydroxyprobe which detects hypoxic/ischemic tissue we also detected increased hypoperfused areas in the subcortical region in young eNOS^−/−^ (4 MO) and eNOS^+/−^ (10 MO) mice which were largely corrected after sodium nitrate (SN) feeding (Fig. 4C). Taken together, we conclude that the hypoperfusion condition upon eNOS deficiency is mild, and chronic in nature, given that we did not detect significantly reduced cerebral blood flow (CBF) in the cortical brain in eNOS-deficient mice at 12-month of age based on Laser Doppler flowmetry (Liao, unpublished data).

In our prior work, we also reported compromised BBB in middle to old-aged eNOS-deficient mice [14]. We speculated that BBB integrity and function may be compromised at a much younger age in eNOS-deficient mice owning to the indispensable role of EDNO signaling in endothelial vascular permeability via modulating VEGF signaling. To detect early BBB leakage, we optimized a fluorescent microscopic method using Evans blue dye (EB, 980 Da) with a very high affinity for serum albumin (67 kDa). Because serum albumin cannot cross the BBB, albumin-bound EB enters the parenchymal tissue in CNS only when the BBB has been compromised, which can be detected in brain sections under a fluorescent microscope using a Rhodamine filter. By this method, we detected massive BBB leakage in eNOS^−/−^ mice beginning at a very young age (6–8-week-old). As shown in Fig. 4D, the eNOS-deficient brains at 3 months of age displayed numerous diffusive spots indicating BBB leakages spanning from frontal to parietal cortical regions compared to no leakage found in littermate control mice. The gradually decreased leakage spots in the five representative angiographs spanning Bregma +2.0 mm to −1.82 mm indicates that leakages started predominantly from an anterior-to-posterior direction (Fig. 4D, E). Indeed, we detected more severe leakage expanded to deeper and posterior areas in older eNOS-deficient mice. Like FITC-hypoperfused lesions, there was no leakage detected in wild-type eNOS mice even at 24 months of age using this methodology (Table 3). Interestingly, mouse brain sections after co-injection of FITC-dextran (2000 kDa) and EB dye displayed largely non- overlapping fluorescent signals in eNOS^−/−^ brain at a young age (data not shown), suggesting that these two early pathological events of non-perfusion and BBB leakage may be independent of each other, likely both resulting from the impaired EDNO signaling. Accordingly, we detected massive upregulation of metalloproteinases 9 (MMP9) in middle age, with MMP9-positive immunosignals largely colocalizing with the EB leakage signals (SFig. 4A–C), indicating breakdown of the endothelial basement membrane. No significant changes in MMP2 expression was detected, and laminin expression was only elevated at 24 months of age (SFig. 4D).

### Sodium nitrate (SN) feeding rescues white matter pathologies and gait behavior in eNOS-deficient mice

Restoration of vascular NO signaling with 1 mM SN in drinking water for up to 10 weeks has been reported to reverse hypertension, systemic hyperlipidemia, and glucose intolerance in 14- to 22-month-old eNOS^−/−^ mice [25]. This prompted us to use this agent to test our hypothesis that feeding eNOS-deficient mice with SN in drinking water at a very young age, starting from 6-week-old for 6 weeks, would prevent the development of the two early pathological events namely hypoperfusion and BBB leakage. The quantitative data presented in Fig. 4D, E, and Table 3 indicate that it is indeed the case; the first cohort of mice that received 6-week-SN feeding resulted in nearly complete prevention of FITC-hypoperfused lesions and BBB leakage. These data encouraged us to further test the possibility of chronic feeding sodium nitrate in drinking water to eNOS-deficient mice at older post-symptomatic age. We fed old eNOS mice with SN feeding for over 3–4 months (Cohort 3 mice, Fig. 4A) and tested them in gait performance; the results demonstrated significant improvement in old eNOS^−/−^ mice (SFig. 5A). Although the SN fed eNOS^−/−^ mice could not perform as well as the eNOS^+/+^ mice at this old age (16 MO), this result is most encouraging with translational value for a potential reversal effect of cortical neuronal connectivity and functions via restoring vascular NO signaling.

The key function of white matter axons is to transmit impulses (action potentials); myelination enables faster impulse transmission [38]. To assess the neurophysiological impact of eNOS deficiency on white matter axons, we examined extracellular compound axon potentials (CAPs) in the CC region. We focused on CC-crossing axons because these axons are mainly layers II/III-originated association axons and more homogenous than other parts of the CC that may also contain passing ascending thalamic axons and descending motor axons [39], and recorded CAPs from Cohort three mice. We detected a significant genotype effect and SN treatment effect on the CAP amplitude of the N1 component: the CC-crossing axon CAP amplitude of the fast N1 component (generated by myelinated axons) was 0.275 ± 0.029 mV for eNOS^+/+^ mice, 0.224 ± 0.026 mV for eNOS^+/−^ mice, 0.122 ± 0.018 mV for eNOS^−/−^ mice, and 0.234 ± 0.035 mV for SN eNOS^−/−^ mice (Fig. 4F). The unmyelinated axon-generated N2 CAP component amplitude (Fig. 4G, H) and also the CAP conduction velocities (data not shown) for N1 and N2 components were not statistically different among the four groups. The most parsimonious interpretation for the reduced CAP N1 amplitude in eNOS-deficient mice is that a significant portion of myelinated CC axons became demyelinated and damaged and even lost in eNOS-deficient mice, leading to a reduction in myelinated axons that are capable of fast impulse conduction. This is consistent with our data from other experiments (Figs. 1 and 2), and with literature data showing hypoperfusion-impaired CC axon myelination [40]. The lack of impairment in the CAP N2 component also indicates that compared with myelinated axons, unmyelinated axons are less vulnerable to the loss of vascular NO signaling. Of note, the CAP N1 component was largely normal in the SN eNOS^−/−^ mice, suggesting that increased vascular NO bioavailability through an exogenous mean can prevent hypoperfusion-induced damages to axon myelination and axonal impulse conduction in the brain.

We also collected additional data from the second cohort of mice showing SN feeding completely prevented the decline of myelin-pathway gene expression (SFig. 2C), and the loss of myelin neurofilaments (SMI-32) in eNOS^−/−^ brains (SFig. 5B). SN feeding also prevented ROS elevation and astrogliosis in selective cortical layers (data not shown). Taken together, chronic SN feeding resulted in significant prevention or improvement in white matter pathologies and functions in eNOS^−/−^ mice.

### eNOS deficiency alters cellular respiration predominantly in white matter at young age

**Fig. 5:**
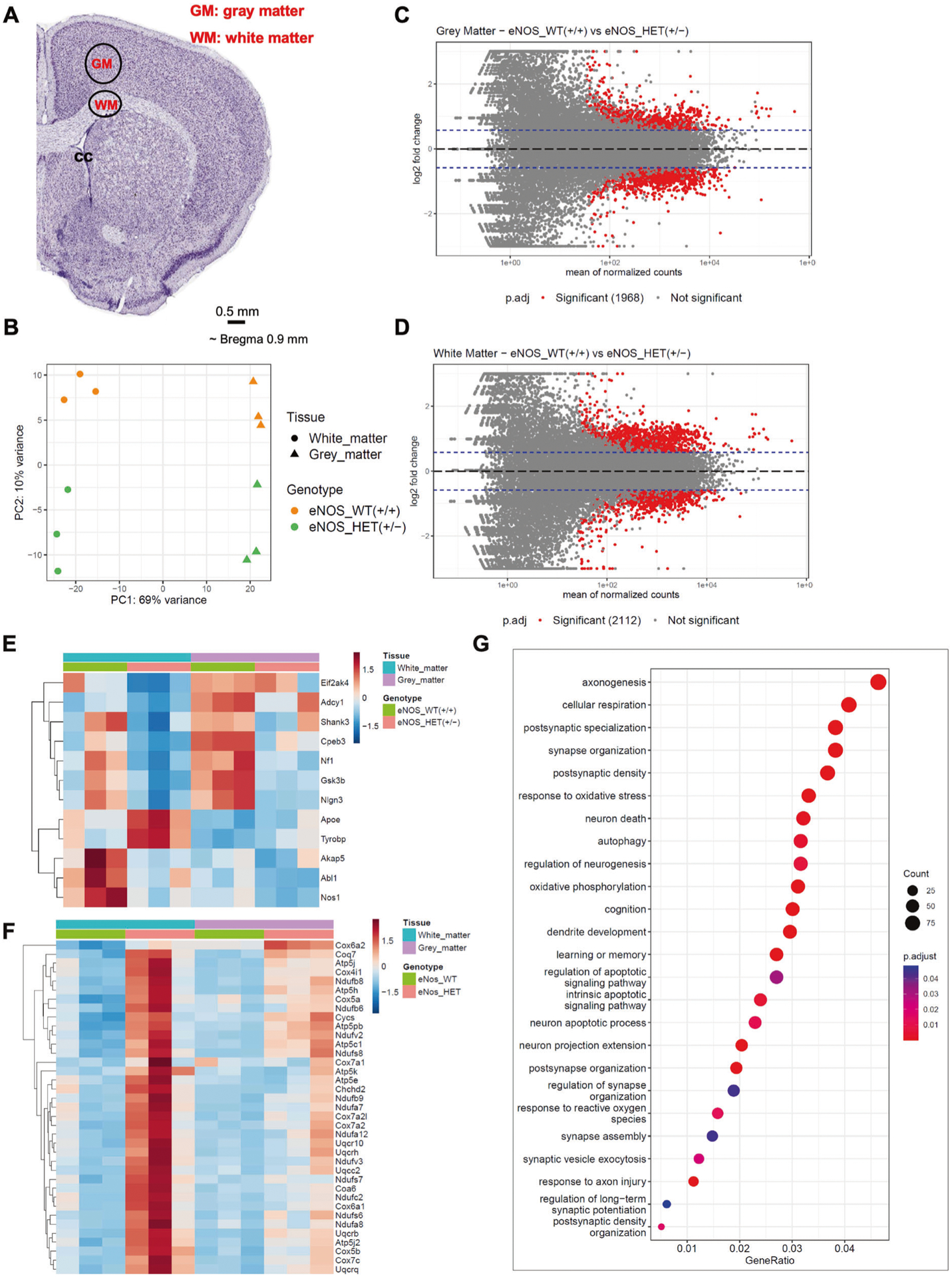
Transcriptional profiling identifies white matter changes in heterozygous eNOS^+/−^ mice. **A** Diagram of the specific brain region tissue for micro punch. **B** PCA plot of the RNA-seq data from white matter and gray matter tissues isolated from the brains of wild-type eNOS^+/+^ and heterozygous eNOS^+/−^ mice. MA plots of the pairwise comparisons between eNOS^+/+^ or eNOS^+/−^, **C** in gray matter and **D** in white matter tissues; log fold changes (LFCs) are plotted against the mean of normalized counts to determine the variance between two treatments in terms of gene expression. Red nodes on the graph represent statistically significant data points i.e., p.adj < 0.05 and LFC > 1.5. Gray nodes are data points that are not statistically significant. Numerical values in parentheses for the significant legend indicate the number of genes that meet the prior condition. Dashed lines indicate the cutoff LFC values. **E** Heatmap showing the alteration of significant genes belonging to gene ontology (GO) term regulation of long-term synaptic potentiation (GO:1900271), in white matter and gray matter tissues isolated from the brains of eNOS^+/+^ and eNOS^+/−^ mice (significant genes p.adj < 0.05 and LFC > 1.5) in the white matter of eNOS^+/−^ compared to eNOS^+/+^. **F** Heatmap showing the progression of significant genes belonging to gene ontology (GO) term cellular respiration (GO:0045333), in white matter and gray matter tissues isolated from the brains of eNOS^+/+^ and eNOS^+/−^ mice (significant genes, p.adj < 0.05 and LFC > 1.5). **G** GO enrichment dot plot showing the number of genes affected in the GO terms related to synaptic plasticity and cognition in white matter tissue isolated from the brains of eNOS^+/+^ and eNOS^+/−^ mice (significant genes, p.adj < 0.05 and LFC > 1.5).

To further elucidate the effects of eNOS deficiency at the transcriptional level, we performed RNA-seq on white matter and gray matter regions dissected from the brains of eNOS^+/+^ and eNOS^+/−^ mice. RNA-seq data analysis followed by the principal component analysis (PCA) showed a clear separation between the tissue type (PC1 – 69% variance) as well as the genotype (PC2—10% variance) (Fig. 5B). We further confirmed the tissue identities by comparing white matter and gray matter regions and found the exclusive enrichment as well as depletion of several tissue specific markers in each of the respective tissue types (i.e., oligodendrocyte markers in white matter vs neuronal markers in gray matter) (SFig. 6A, B).

Further analysis with DESeq2 revealed the differential expression of 1968 genes in gray matter (Fig. 5C) and 2112 genes in white matter (Fig. 4D) significantly (p.adj < 0.05 and LFC > 1.5) between eNOS^+/+^ and eNOS^+/−^ genotypes, confirming the effect of eNOS haploinsufficiency. From the set of significant genes that were revealed in white matter tissue that were differentially expressed between eNOS^+/+^ and eNOS^+/−^, we identified significant alteration pattern in genes related to the gene ontology (GO) biological process terms ‘regulation of long-term synaptic potentiation’ (GO:1900271) and ‘synapse assembly’ (GO:0007416) (SFig. 6C) that were driven primarily in gray matter (Fig. 5E), and cellular respiration (GO:0045333) (Fig. 5F), predominantly progressing in white matter. GO enrichment was done on these significant genes, and the dot plot shows the alteration of GO terms related to cellular respiration, oxidative phosphorylation, response to oxidative stress as well as synaptogenesis, neurogenesis, axonogenesis and neuronal apoptotic process (Fig. 5G) indicating impaired mitochondrial function underlying the white matter pathology in this model. Additionally, SN feeding corrected these altered genes to large degrees (SFig. 6D).

### eNOS deficiency induces upregulated BMP4 signaling at young age

**Fig. 6:**
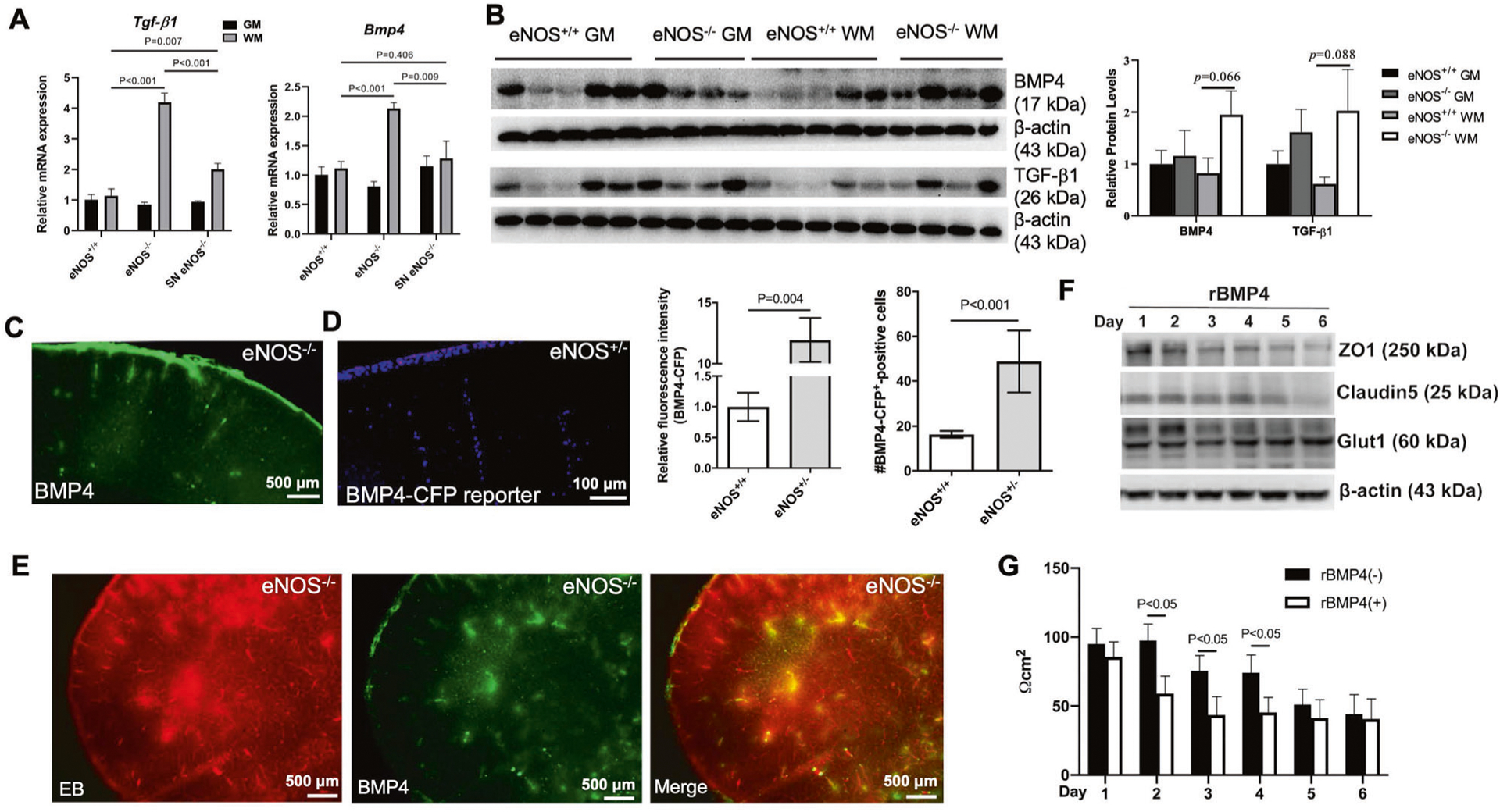
Potential negative impact of the aberrantly upregulated BMP4 signaling upon eNOS deficiency on BBB. **A** qPCR graphs showing increased expression of the two genes of the large transforming growth factor TGFβ family: *Tgf-β1*: transforming growth factor-β1; *Bmp4*: bone morphogenetic protein 4. Data are presented as mean ± s.d.m., normalized by G*apdh* gene expression. *N* = 3–4 mice/group. **B** Representative western blots and graph of semi-quantitative analysis based on densitometry. **C** Microscopic image of BMP4 immunosignals taken from eNOS^−/−^ brain (5 MO). **D** Representative fluorescent images taken from the forebrain of BMP4-CFP reporter mouse on an eNOS^+/−^ background (5 MO) using wide DAPI filter. Quantification graphs were based on the BMP4-CFPepositive cells and on the fluorescent intensity of the positive cells, respectively (*N* = 3 mice/genotype, comparison between eNOS^+/+^ and eNOS^+/−^). **E** Microscopic image of BMP4 immunosignals on the same brain section taken from eNOS^−/−^ brain for Evans blue angiography. **F** Transendothelial electrical resistance measurement (TEER values) of the bEnd.3 cells (monolayer BBB model) treated with recombinant rBMP4 (25 ng/mL; mean ± s.e.m; *N* = 3 independent experiments). **G** Representative western blots of tight junction marker proteins of rBMP4-treated bEnd.3 cells (*N* = 3 independent experiments).

Members of transforming growth factor-β superfamily, which include the largest members of bone morphogenetic proteins (BMP1–9), are implicated in aging, cardiovascular diseases, AD and VCID [41]. BMP4 signaling has been widely reported as a key negative regulator of brain development, adult hippocampal neurogenesis, astrogliogenesis, and oligodendrocyte differentiation [42]. BMP4 is upregulated in the brains of patients with vascular dementia as well as in the hypoperfused mouse brains undergoing bilateral common carotid artery stenosis [43]. Consistently, we also detected upregulated *Bmp4* gene expression on the isolated microvessels from eNOS^−/−^ mouse brain at young age, along with *Tgf-β1* gene (Fig. 6A). Their protein levels appeared to be higher, more selectively in the white matter tissue than in gray matter (Fig. 6B). Increased immunohistochemistry signals of BMP4 upon eNOS deficiency indicated a vascular source (Fig. 6C) which was confirmed by an alternative approach of using BMP4 reporter mice with a fusion of the cyan fluorescent protein gene (CFP) under mouse BMP4 promoter [44]. Both the CFP fluorescent signals and the number of nuclear BMP4^+^ cells were quantified showing upregulated BMP4 expression from the descending arterioles in the frontal-parietal cortex in eNOS-deficient mice at a young age (Fig. 6D). Immunohistochemistry of BMP4 on the brain sections processed for Evans blue angiography revealed nearly complete colocalization of the BMP4 immunosignals with the EB extravasation (Fig. 6E), indicating that BMP4 expression and secretion were induced on the vasculature with disrupted BBB upon eNOS deficiency. We then used an in vitro cellular model of BBB to investigate the impact of upregulated BMP4 signaling via a time-course study upon treatment with recombinant rBMP4 protein. Similar functional outcome results were obtained from using a monolayer model of cultured endothelial bEnd.3 cells or a co-culture model of bEnd.3 and C8-D1A astrocyte cells with bEnd.3 cells on the top or in the bottom of transwells. Representative data on the expressional changes of tight junctional marker proteins and the BBB integrity (i.e., TEER values) are presented in Fig. 6F, G, showing disrupted BBB by rBMP4 treatment.

## DISCUSSION

In humans, brain white matter is evolutionarily expanded, comprising half of the brain mass with vast numbers of axons, reaching an astonishing total length of 170,000,000 m in young men. These white matter axons transmit electrical signals and enable communications among different cortical and subcortical areas that eventually produce brain function and cognition. Over the past decade, white matter is increasingly recognized as equally critical as the cerebral cortex and hippocampus for cognition. This is not surprising given the fact that white matter tracts form an indispensable component of the complex neural connectivity that supports neurobehavioral operations. Nowadays, there is compelling evidence supporting that white matter lesions disturb and probably directly contribute to cognitive dysfunctions [45].

Myelination, a process that continues for decades in the human brain is crucial for information processing by regulating the velocity of axonal impulse conduction between distant cortical and subcortical regions. It is now well recognized that the process of myelination can be modified by experience [46, 47]. Compelling evidence collected from aging and AD brains suggests that demyelination within the cerebral white matter is an upstream event that initiates amyloid deposition and then tau as later end products comprising cortical pathology in AD [48]. Although this “myelin model” was proposed initially in the context of AD, the same theory may be generalized to other neurodegenerative and psychiatric conditions including vascular dementia. Thus far, this model has been largely supported by compelling evidence including recent genetic data [19], which is in line with the concept of white matter dementia formed two decades ago [49] to highlight the importance of white matter dysfunction to cognitive decline. The tight association between the severity of dementia and white matter lesions provides additional strong support to this theory. Since dementia has been most extensively studied in AD, our current therapeutic development towards dementia is dominated by anti-Aβ/tau strategies with all clinical trials ending in failure. If the myelin model proves to be correct as a unifying theory for all types of dementia including cortical, white matter and subcortical dementia, it would be instrumental in redirecting our focus from amyloid and tau to myelin-oriented preventive and treatment interventions. Therefore, a deeper understanding of the mechanisms of demyelination/remyelination at both cellular and molecular levels would be fundamentally important in designing novel targeting strategies long before the onset of cognitive dysfunction. This relies not only on the knowledge gained from clinical neurology (e.g., neuroimaging and neuropathology) to identify early white matter pathology but also from various representative experimental models of dementia conditions to validate the earliest white matter mechanisms that drive cognitive decline.

### Experimental models of vascular dementia

This report presents evidence that genetic eNOS-ablation spontaneously induces chronic hypoperfusion, BBB leakage, elevated ROS/astrogliosis, myelin loss, and axonal pathology as well as cortical neurodegeneration, impaired associative recognition memory and gait imbalance in mice. All these phenotypes are considered the essential components as potential contributing mechanisms to dementia, based on both clinical [18, 20–24] and experimental [41] evidence. Clinically, dementia has been most widely studied in AD type so-called cortical dementia, the other two types of dementia (i.e., subcortical dementia, often seen with stroke patients), and white matter dementia are featured by distinct core cognitive dysfunctions, especially at the early stages of dementia, likely due to different white matte lesions [45]; of note, these features become indifferent as neuropathologies progress to late stages, all three types of dementias become indistinguishable in the end. Again, these clinical notions underscore the paramount importance of studying white matter myelin mechanisms using representative experimental models.

In addition to the transgenic/knock-in rodent models based on mutant familial AD genes (e.g., APP, PS1, and tau) in which white matter dysfunction was occasionally reported, there are currently more than a dozen of rodent models that develop major features of human dementia with the vascular origin [50]; none of a single model recapitulates all features of human dementia. Chronic cerebral hypoperfusion has been suggested to be a key mechanism leading to dementia [13]. Over the last decade, bilateral common carotid occlusion/stenosis (BCAS) has been the most widely used chronic hypoperfusion model. This BCAS surgery in both rats and mice induces several major white matter changes including reduced parenchymal CBF, oligodendrocyte loss, glial activation, and disrupted clearance of intracerebral fluid and BBB, and cognitive impairment [51]. However, sizable intrinsic variance in cerebral hypoperfusion with CBF dropping too suddenly and too severely after occlusion surgery is one major shortcoming of this type of surgical model. Moreover, all pathological events occur within 2–3 months, making the model difficult to consider the aging component. In our opinion, the spontaneously developed white matter and cognitive phenotypes in an age-dependent manner in the mouse model based on eNOS deficiency (both heterozygous eNOS^+/−^ and eNOS^−/−^ knock-out mice) makes these mice ideal for studying white matter mechanisms, linking chronic hypoperfusion and demyelination directly to neurodegeneration.

### White matter changes, including myelin loss, and axonal pathology

The largest white matter tract, the corpus callosum (CC), is particularly vulnerable to stress. This region has been extensively examined and reported for various pathological changes in the brains of healthy elderly individuals, AD, multiple sclerosis and vascular dementia. Although we observe CC atrophy in eNOS^−/−^ brain at very old age, we detect demyelination in cortical and CC regions, but not striatum, at a much younger age (starting from 7 MO). The mechanism for CC atrophy is unclear, presumably resulting from white matter damage.

Maintenance of the integrity of the myelinated axons and the functional coupling between axons and the responsible glial cells (i.e., oligodendrocytes) are well-known to be critical for action potential propagation and thus for regulating efficient neuronal connectivity. Axonal dysfunction in white matter disorders is well recognized to confer a worse prognosis than that implied by myelin damage alone [50]. White matter axon-glial integrity has been reported to be disrupted in mice by mild cerebral hypoperfusion in an experimental model (BCSA) [51]. We used the pan-antibody SMI-32 to visualize the distribution of mixed neurofilaments in eNOS^−/−^ brain at middle age and observed significantly reduced immunosignals in multiple regions, including CC and subcortical areas (SFig. 5B); the thinner and more “chaotic” staining pattern displayed in the eNOS^−/−^ brain also indicated reduced axonal diameters and aberrantly sprouting axons derived from cortico-cortical fibers, predicting the loss of synaptic circuitry underlying the basis of memory. Moreover, the selectively reduced values in the extracellular compound axon potentials (CAPs) N1 amplitude in eNOS-deficient mice, but not in the unmyelinated axon-generated N2 CAP component amplitude N2, indicate vulnerability of the myelinated axon upon hypoperfusion conditions. Taken together, we conclude that both the integrity of the myelin sheath (assessed by MBP) and integrity of axons (assessed by SMI-32), as well as the functionality of myelinated axons (assessed by CAP N1 amplitude) are compromised upon eNOS deficiency. We speculate that these axonal pathologies occur early, before the neuronal cell body pathology. Beside the data present from old mice (Fig. 4F–H), we have preliminary data indicating that CAP N1 amplitude in eNOS^−/−^ mice started to decline at 9 months of age. We also observe demyelination at a younger age in eNOS^−/−^ mice (7 MO) while no cortical layer pyramidal neurodegeneration is detected at this age. Additionally, the demyelination is more severe in cortical outer layers than in cortical inner layers, consistent with the literature data that the outer cortical layers II/III are most vulnerable to hypoperfusion in the clinical specimen of both early demented AD and post-stroke brains [52, 53].

### Chronic hypoperfusion and BBB dysfunction

We have collected evidence to identify hypoperfusion and BBB leakage as the two earliest pathological events in eNOS^−/−^ brains from newly weaned mice (6 weeks of age) via FITC and EB angiographies, respectively. We discovered a general pattern through systemic examination of the coronal brain sections from these angiographies for these two events, both expanding from the anterior to the posterior brains in an age-dependent manner. While FITC-dextran non-perfused lesions occur predominantly in the frontal parietal cortical area, Evans blue leakages are present globally in both dorsal and ventral cortical areas. The overall “anterior-to-posterior” hypoperfusion gradient is reminiscent of the clinical hypoperfused human brains correlating with various stages of dementia [54, 55]. For example, dysexecutive individuals often display significant hypoperfusion in the left superior, medial frontal, and cingulate cortex. In contrast, the amnestic mild cognitive impaired individuals show significant hypoperfusion in the left hippocampus, para-hippocampal gyrus, and frontal-parietal-temporal areas [56].

Perhaps limited by the nature of the two angiographic methods used in this study, we could not determine which of these two events occurs first. Given the different angiographic profiles from FITC-dextran and EB, we posit that they are independent events, both resulting from impaired EDNO signaling. Indeed, we observe non-overlapping fluorescent signals from the brains receiving a co-injection of both dyes. However, the main question remains of how these initial pathological events lead to the subsequent white matter pathologies. White matter is long speculated to be most vulnerable to chronic hypoperfusion due to intrinsic lower microvessel density [10, 24]. Despite a recent report excluding BBB leakage as an invariant feature of the white matter lesions based on case studies from the genetic paradigm of small vessel disease so-called cerebral autosomal dominant arteriopathy with subcortical infarcts and leukoencephalopathy (CADASIL) [57], more experimental evidence supports a prevailing view that disrupted BBB can contribute directly to white matter changes. For example, using a model of pericyte-deficiency, white matter pathology, and impaired executive functions were detected. In this model study, oligodendrocyte degeneration was suggested to be caused by an accumulation of toxic blood-derived fibrin(ogen) deposits and blood-flow reductions; the latter further leads to a loss of oligodendrocytes, and thus myelin, and axonal structure and function [58]. Our data are consistent with the observations from this study: both the accumulation of fibrin(ogen) deposits are detected in the eNOS model as previously reported [14, 41]. We also collected preliminary evidence showing reduced pericytes in the eNOS model at young age (Liao, unpublished data).

### ROS, astrogliosis and white matter neuroinflammation-what causes demyelination?

Astrocytes contact blood capillaries with their end feet and are therefore an important part of the blood–brain interface, presenting differences between gray (GM) and white matter (WM). Interestingly, astrocytes exert different properties of protoplasmic and fibrous subtypes in GM and WM, respectively. Therefore, it is likely that these different astrocyte subtypes contribute to the observed differences in BBB properties in GM and WM. We detect marked BBB leakage in eNOS^−/−^ brain at a very young age, followed by increased expression of GFAP-positive reactive astrocytes in the CC region and selected cortical layers II/III and V/VI by mid-age. Given the exclusive upregulated pro-inflammatory cytokine gene expression in the CC white matter region, we reason that the increased astrogliosis in the CC region likely contribute directly to oligodendrocyte degeneration and demyelination while the sparse presence of the GFAP-astrocytes in the cortical areas surrounding the degenerated layer II/III and V/VI pyramidal neurons likely reflect the well-recognized role of astrocyte in tissue repair upon injury. The precise astroglial mechanisms in various pathogenic stages of the white matter changes in the eNOS model will be further investigated at cellular and molecular levels. Our finding of the upregulated BMP4 signaling at a very young age (4–5 MO) upon eNOS deficiency opens up a new window of investigating impaired signaling of the TGFβ family as a potentially crucial glial-vascular mechanism of the white matter changes in the eNOS model. We speculate that the well-known paracrine actions of BMP4 signaling could directly trigger white matter degeneration via promoting astrogliosis and simultaneously interfering with oligodendrocyte differentiation, and maturation. The data based on the in vitro BBB models indicate that upregulated BMP4 signaling can disrupt BBB integrity and induce inflammatory pathway genes in endothelium (SFig. 7). Our preliminary data also suggest that pericytes are the major source of upregulated BMP4, and it remains to be determined for the paracrine roles of BMP4 signaling on the glia-vascular coupling. Although we did not detect significant increase in Iba-1 positive microglia in eNOS-deficient mouse brain by mid-age (12 MO), we did detect increased TGF-β1 primarily in microglial at a young age (Liao, unpublished data). Potentially differential roles of microglia as to astrocyte in the development of the white matter pathologies in the eNOS model warrant further studies. A novel role of microglia in modulating neurovascular coupling function and blood flow has been suggested in a recent report [59]. An important question remains of how an impaired EDNO signaling induces TGF-β1 and BMP4 signal transduction in potentially cell-type specific manner.

### Mitochondrial dysfunction as a major trigger to white matter pathology?

In brain, both neuron and oligodendrocytes represent the most energy-demanding cell types. Mitochondria as the major source for generating ATP for cellular functions have been identified as one early pathological event during the development of cognitive impairment/dementia [60]. NO signaling has long been established to be crucially linked to mitochondrial performance (e.g., oxidative phosphorylation) and to modulating neurovascular coupling [60, 61], traditionally attributed to the NMDAR-nNOS coupling in neurons upon neuronal activity to regulate regional CBF [62]. Under baseline conditions, vascular NO signaling is believed to play a more significant role in modulating CBF and maintaining cerebral vascular homeostasis. Neurovascular coupling is impaired in eNOS-deficient mice at mid-age (14 MO) based on a recent report [63]. Our bulk RNAseq analysis reveals that the expression of the cellular respiratory pathway genes is reduced at a young age in the eNOS-deficient model more selectively in white matter, suggesting impaired mitochondrial functions. As illustrated in SFig. 8, we speculate that the impaired mitochondrial function is a result of initial endothelial dysfunction induced by hypoperfusion and the disrupted BBB which can form a vicious cycle to further compromise the glia-vascular coupling, as supported by previous work from an independent group [64], and thus affect neurovascular functions owning to the elevated ROS and the subsequently induced neuroinflammation.

### NO-based therapy—inorganic nitrate/nitrite

In humans, EDNO plays a key role in regulating basal CBF via vasodilation of cerebral vessels, and aberrant biogenesis of NO is associated with arterial stiffness, hypertension, atherosclerosis in patients with cardiovascular diseases, AD and vascular dementia patients [41]. Our data demonstrate that impaired EDNO signaling can first lead to cerebral local hypoperfusion and the impairment of the BBB in frontal forebrain, subsequently to white matter changes and neurodegeneration from cortical to hippocampal regions. Developing NO-based therapeutics thus represents a viable strategy for treating white matter dementia.

Since the seminal discoveries of NO as the most crucial endothelial-derived relaxing factor, and subsequently, the biology of nitrite anion (i.e., the “nitrate-nitrite-NO pathway”) [65], and the developing NO-based therapeutics have been actively pursued. Several mechanisms probably contributed to the decrease in NO bioavailability, including defects in the cascade of NO generation (e.g., eNOS activity) and reduced NO donors. Thus far there are only three agents that are approved by FDA for use in treating acute angina by organic nitroglycerine, NO inhalation for pulmonary hypertension in neonates, and phosphodiesterase inhibitors to prevent the breakdown of the downstream cyclic guanosine monophosphate/cGMP (i.e., second messenger of NO) [66]. Although the first line cholesterol-lowering drugs (i.e., statins) also demonstrate a potential to enhance the bioavailability of EDNO, principally by up-regulating and activating eNOS [67, 68]; convincing clinical data is still lacking [69, 70]. Importantly, like L-arginine supplementation based on the discovery of it being the physiological substrate of eNOS, statins belong to the same category of NO-enhancing strategies requiring functional NOS system as a prerequisite. In theory, using strategies independent of a functional NOS system to increase the bioavailability of NO production is more ideal.

Historical evidence suggests that using dietary supplements with natural products represents a safest strategy [71]. Numerous experimental evidence indicates that nitrite anion represents an important reservoir of NO bioequivalents to increase tissue NO levels during pathophysiological states in which NO synthesis from the eNOS-mediated enzymatic pathways are unavailable; nitrite anion production and distribution throughout the body can act in an endocrine manner to augment NO bioavailability, as demonstrated in a prior study [72]. Administration of nitrate or nitrite to humans and rodents clearly led to an increased NO-like bioactivity from numerous studies [66]. Feeding eNOS-deficient mice with oral sodium nitrite or sodium nitrate has also been demonstrated by independent groups to restore vascular NO signaling and vascular homeostasis [25, 73]. Our data not only reproduces these observations but also expands to the BBB and white matter beneficial effects (Fig. 4). These promising results provide additional mechanistic insights to the NO-based therapy, mechanistically, highlighting the paramount importance of the EDNO signaling in white matter health.

## CONCLUSIONS

White matter lesions can directly contribute to cognitive dysfunctions via dis-coupled glia-vascular functions. Since we observe hypoperfusion and BBB breakdown before myelin loss in white matter, we speculate that the compromised BBB with vascular injury, along with oxidative stress and astrogliosis, all contribute to the subsequent white matter change. Moreover, we also detect selective neurodegeneration in the cortical layers (II/III and V/VI), likely due to demyelination. The chronically evolving nature of the spontaneous model of mild hypoperfusion in eNOS-deficient mice will allow us to tease out the sequential pathological events in a future longitudinal study, at both molecular and cellular levels. These mice offer a unique opportunity to generalize and further validate the “myelin model” in a mouse system without being compounded by the amyloid and tau as dominating factors. We thus conclude that eNOS-deficient mice may represent an ideal spontaneous model for studying the earliest events leading to white matter changes. Moreover, it would be useful for future testing drug candidates for therapeutic use via targeting novel/specific vascular mechanisms contributing to VCID and AD.

## Supplementary Material

supplementry material

## Figures and Tables

**Table 1.** Summary of the antibodies used in immunohistochemistry and in Western blots.

Specificity	Species	Source/Catalog	Dilution
MBP	Rabbit	Abcam/ab40390	1:1000
NF-200	Mouse	Sigma/NO142	1:1000
CC1	Mouse	Millipore/OP-80–100UG	1:500
Oligo1	Mouse	Santa Cruz/sc-166256	1:500
SMI-32	Mouse	Sigma/NE1023	1:1000
Neu N	Rabbit	Abcam/ab177487	1:500
GFAP	Mouse	Sigma/G3893	1:1000
S100β	Rabbit	Abcam/ab41548	1:1000
Iba-1	Rabbit	Wako/019–19741	1:1000
Aqua4	Rabbit	Novus/NBP-97928	1:1000
GLUT1	Rabbit	Invitrogen/PAL-46152	1:500
Laminin	Rabbit	Sigma/L9393	1:500
MMP9	Mouse	Millipore/MAB13415	1:500
MMP2	Mouse	Invitrogen/MA5–13590	1:500
HIF-1α	Rabbit	Cell Signaling/3716s	1:500
HSF1	Rabbit	Cell Signaling/4356s	1:500
MOG	Mouse	Millipore/3307816	1:500
PLP1	Rabbit	Abcam/ab28486	1:1000
TGF-β1	Mouse	Santa Cruz/sc-130348	1:1000
BMP4	Mouse	Millipore/MAB1049	1:1000
ZO-1	Rabbit	Invitrogen/61–7300	1:1000
Claudin5	Mouse	Invitrogen/UB280529	1:1000
β-actin	Mouse	Novus/NB600–501	1:5000

**Table 2.** Summary of the oligonucleotide primers used in quantitative PCR analysis.

Target gene	Forward primer	Reverse primer
*Il-1β*	AAATGCCTCGTGCTGTCTGACC	CTGCTTGAGAGGTGCTGATGTACC
*Il-6*	AGTTGCCTTCTTGGGACTGA	TCCACGATTTCCCAGAGAAC
*Tnf-α*	CCCTCACACTCAGATCATCTTCT	GCTACGACGTGGGCTACAG
*Mbp*	GGAAGGCAGGTGATGGTTGA	ACACTGGAGGGCAAACACTC
*Plp1*	CTTCCCTGGTGGCCACTGGATTGT	CCGCAGATGGTGGTCTTGTAGTCG
*Mog*	TCCATCGGACTTTTGATCCTCA	GCTCCAGGAAGACACAACCA
*Mag*	GGTGTTGAGGGAGGCAGTTG	CGTTGTCTGCTAGGCAAGCA
*Tgf-β1*	TGGAGCAACATGTGGAACTC	GTCAGCAGCCGGTTACCA
*Bmp4*	ATTCCTGGTAACCGAATGCTG	CCGGTCTCAGGTATCAAACTAGC
*Icam1*	TTCACACTGAATGCCAGCTC	GTCTGCTGAGACCCCTCTTG
*Vcam1*	ATTTTCTGGGGCAGGAAGTT	ACGTCAGAACAACCGAATCC
*Mmp2*	TAACCTGGATGCCGTCGT	TTCAGGTAATAAGCACCCTTGAA
*Mmp9*	ACGACATAGACGGCATCCA	GCTGTGGTTCAGTTGTGGTG
*Vegfa*	GTTGCCTAGTGGGTGGATCT	GCTACCCATCCAGCCTGTT
*Nox1*	CTTGCACCGATTGCTTTTTAT	CATTAGATGGGTGCATGACAA
*Nox3*	GGAACGACGAATTCAAGCAG	CACAGAAGAACACGCCAATG
*Nox4*	GCAGATTTACTCTGTGTGTTGCAT	TCCCATCTGTTTGACTGAGGT
*Gapdh*	GCAAATTCAACGGCACAG	CTCGCTCCTGGAAGATGG

**Table 3.** Summary of the degrees of non-perfusion lesions based on FITC angiography (Cohort 2).

Bregma (mm)	eNOS^+/+^	eNOS^−/−^	SN eNOS^−/−^
+2.0	A: (−)	A: (++ to +++), bilateral	A: (− to +), bilateral
	B: (−)	B: (+), unilateral	B: (− to +), unilateral
+1.4	A: (−)	A: (++), bilateral	A: (− to +), unilateral
	B: (−)	B: (++), unilateral	B: (−)
+0.8	A: (−)	A: (++), uni- and bilateral	A: (− to +), bilateral
	B: (−)	B: (+), uni- and bilateral	B: (−)
+0.2	A: (−)	A: (++ to +++), bilateral	A: (− to +), unilateral
	B: (−)	B: (+), unilateral	B: (−)
−0.6	A: (−)	A: (+ to ++), bilateral	A: (− to +), bilateral
	B: (−)	B: (+ to ++), uni- and bilateral	B: (− to +), uni- and bilateral
−1.0	A: (−)	A: (++), bilateral	A: (− to +), bilateral
	B: (−)	B: (+ to ++), unilateral	B: (−)
−1.6	A: (−)	A: (+ to ++), bilateral	A: (− to +), unilateral
	B: (−)	B: (++), unilateral	B: (−)
−2.2	A: (−)	A: (++), unilateral	A: (− to +), bilateral
	B: (−)	B: (+ to ++), unilateral	B: (−)

A: dorsal cortex.

B: ventral cortex.

*N* = 3 mice/group at mid-age (11–12 MO) with exception for the eNOS^+/+^ group (2/3 at 12 MO and 1/3 at 24 MO).

Scoring system: (+) 1–2 lesions; (++) 3–4 lesions; (+++) > 4 lesions at larger size.

## Data Availability

Raw and processed RNA-seq data used in this study are available at the Gene Expression Omnibus under the accessions GSE207960, GSE207961 and GSE207693. Codes used in this study are available from the authors MCK and WZ upon request.
