# Supplementary figures and images for: White matter damage as a consequence of vascular dysfunction in a spontaneous mouse model of chronic mild chronic hypoperfusion with eNOS deficiency
Source: Mol Psychiatry. Author manuscript; Available in PMC 2022 Dec 12. (PMC9734049; doi:10.1038/s41380-022-01701-9)

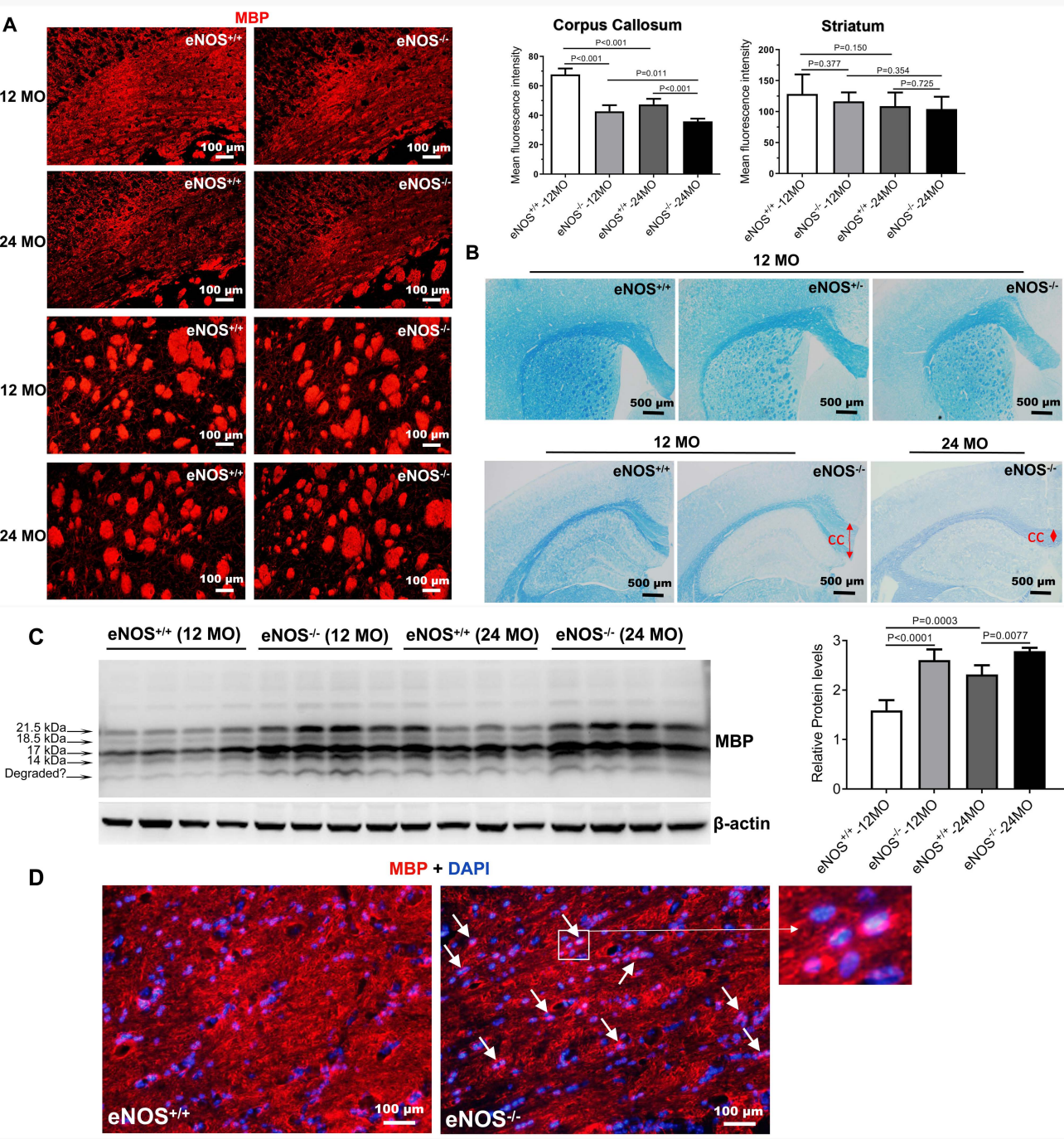

**A****PLP****B****HIP**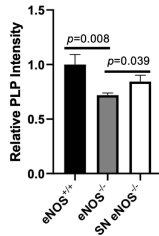**C****MOG**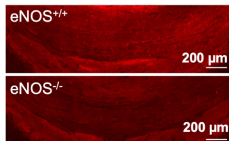**D**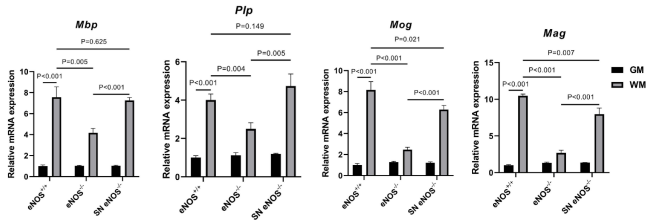

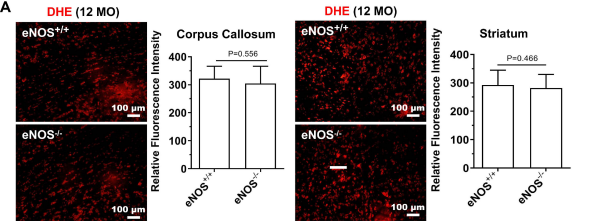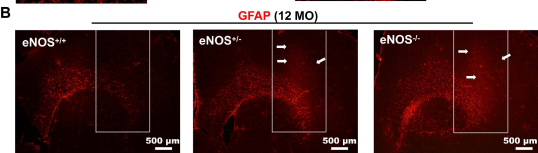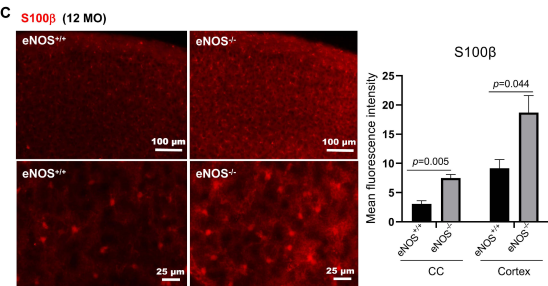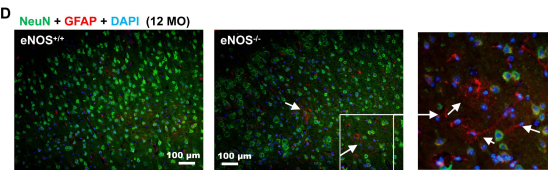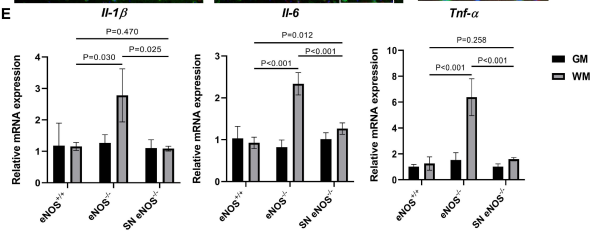

**A**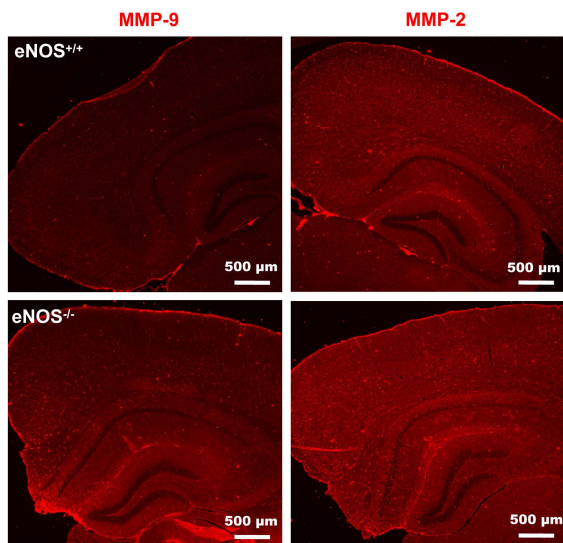**B**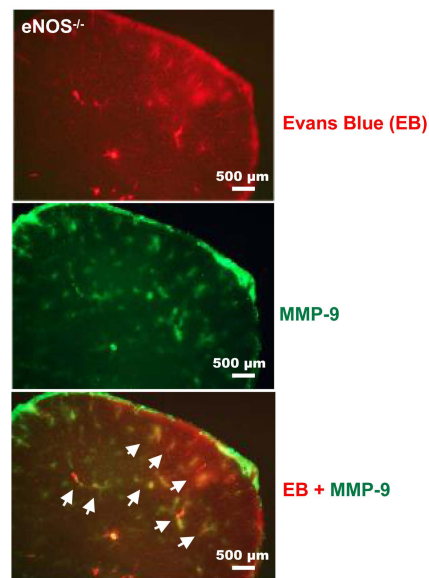**C**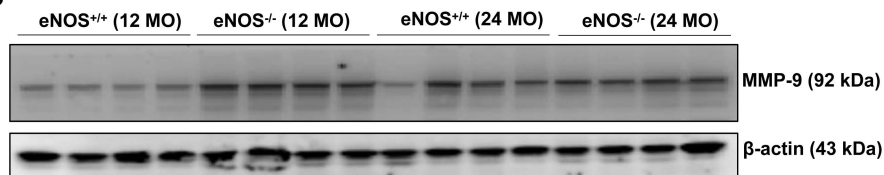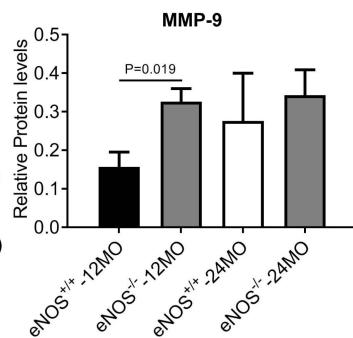**D**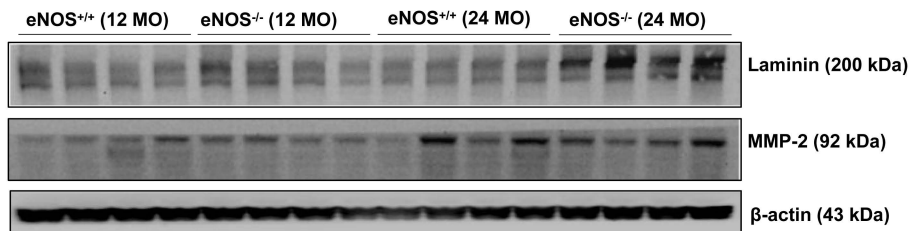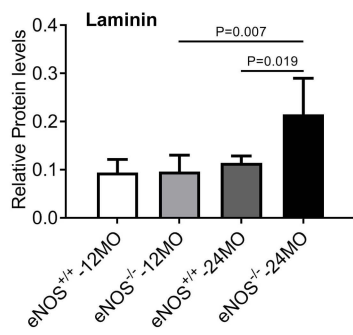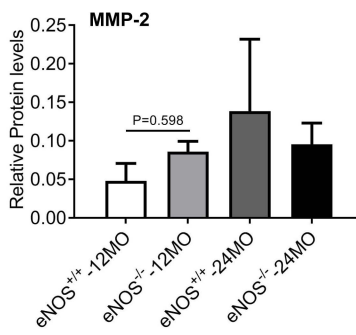

**A**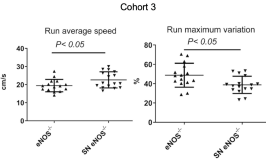**B**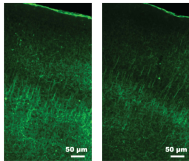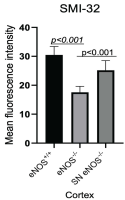

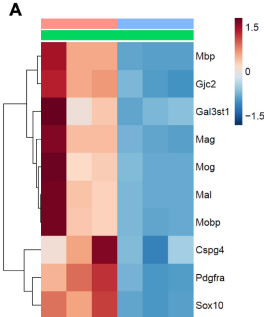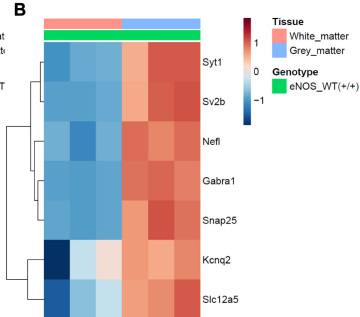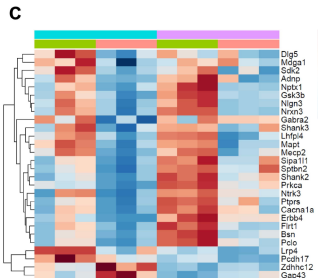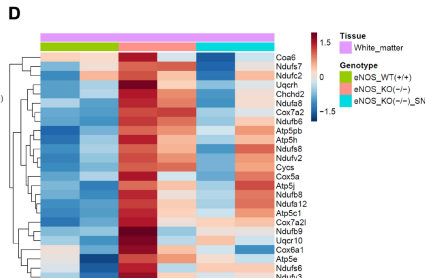

**A**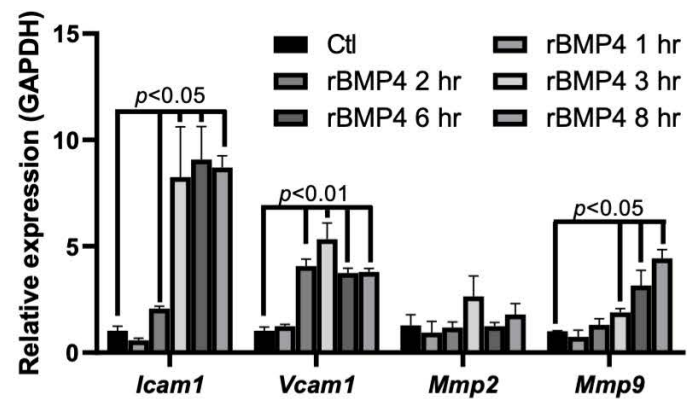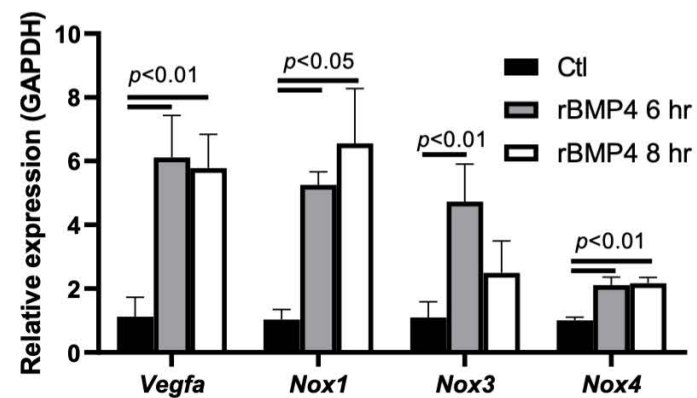**B**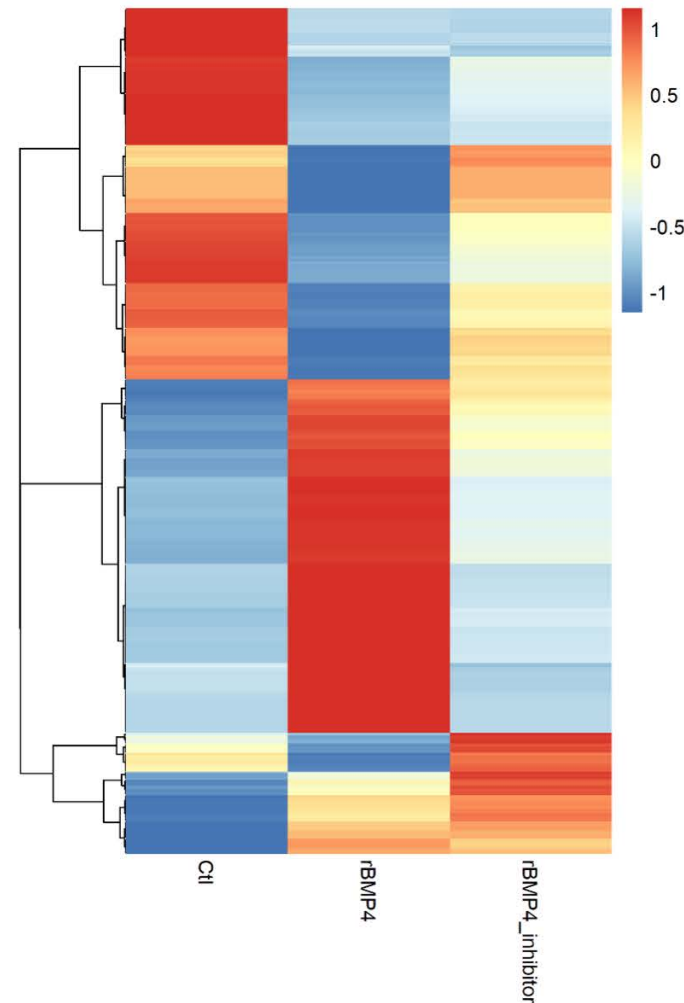**C**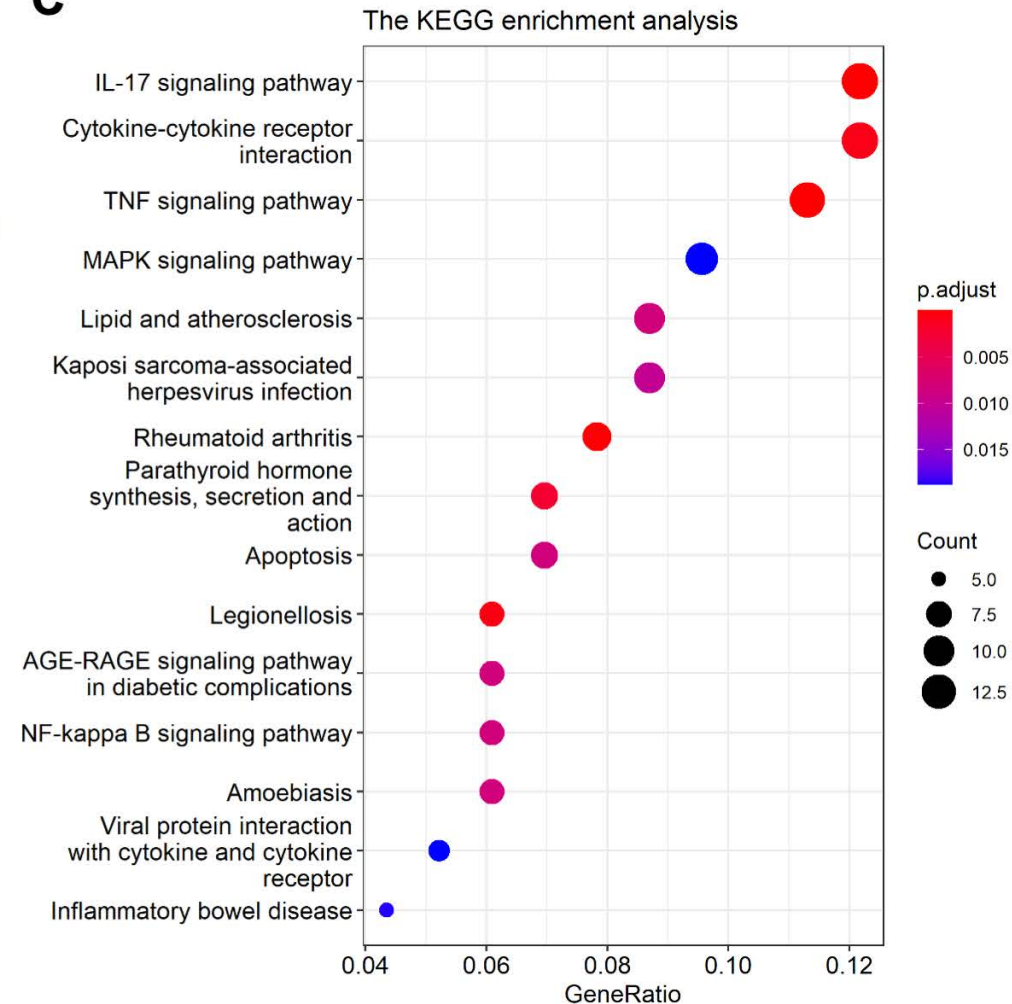

# eNOS-deficient Mice Impaired Vascular NO Signaling

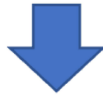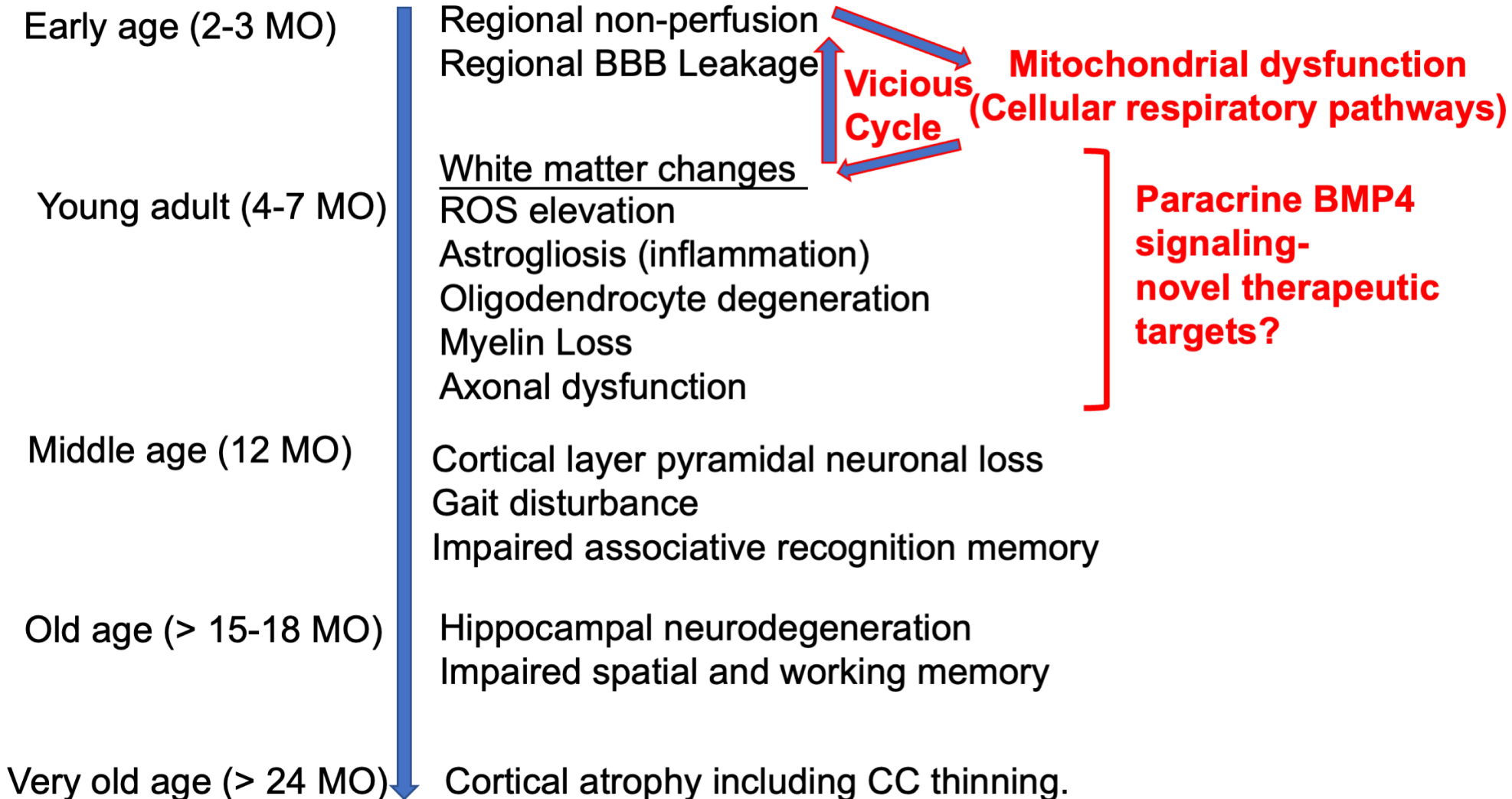

Supplement: supplementry material [file NIHMS1834417-supplement-supplementry_material.pdf]
