# Supplementary material for: White matter damage as a consequence of vascular dysfunction in a spontaneous mouse model of chronic mild chronic hypoperfusion with eNOS deficiency
Source: Mol Psychiatry. 2022 Aug 10;27(11):4754–69. doi: 10.1038/s41380-022-01701-9 (PMC9734049; doi:10.1038/s41380-022-01701-9)
Supplement: Supplementary file 1 [file 41380_2022_1701_MOESM1_ESM.pdf]

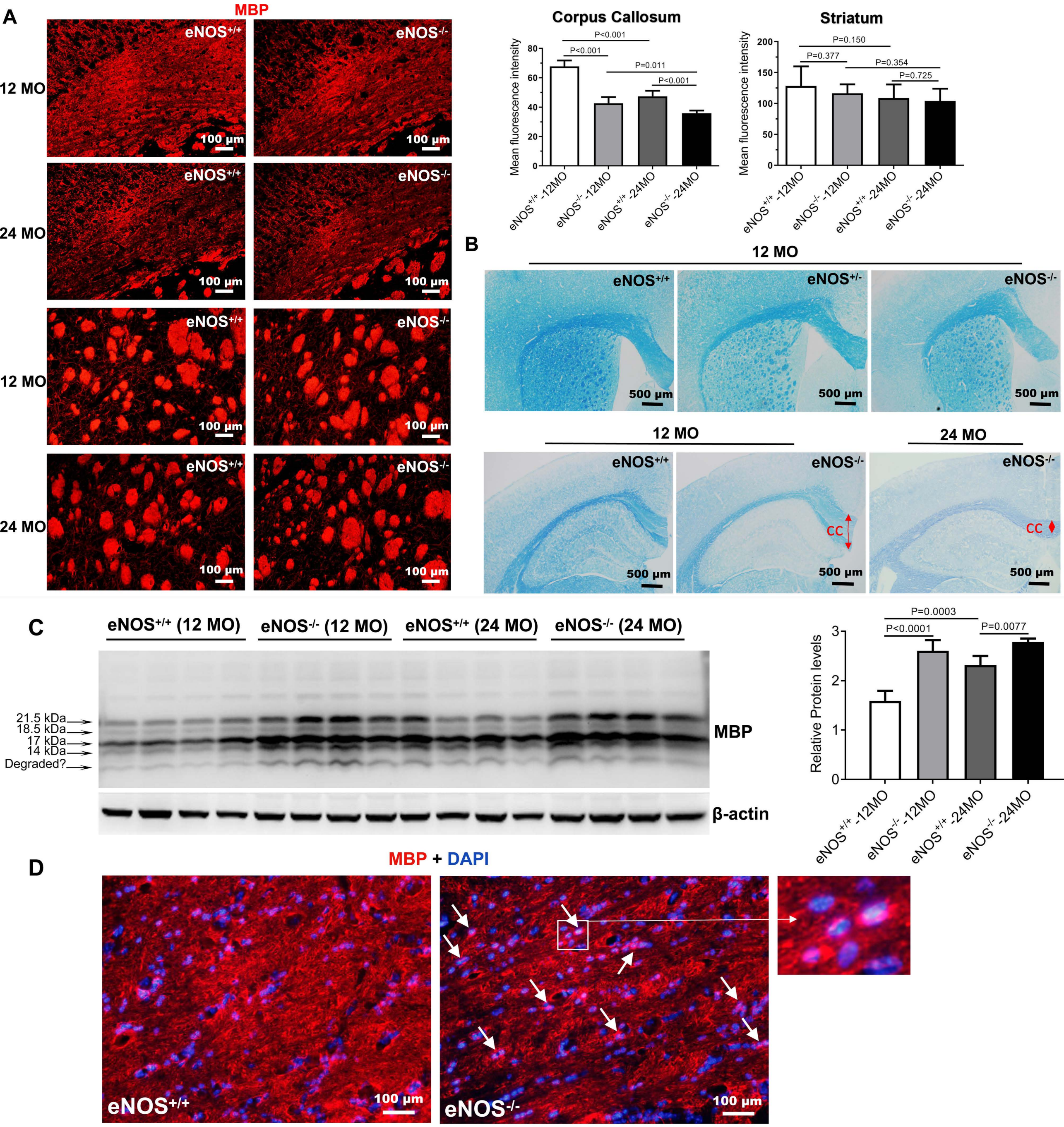

A
MBP
eNOS+/+
eNOS-/-
12 MO
24 MO
100 μm
Corpus Callosum
Mean fluorescence intensity
P<0.001
P<0.001
P=0.011
P<0.001
eNOS+/+ -12MO
eNOS-/- -12MO
eNOS+/+ -24MO
eNOS-/- -24MO
Striatum
P=0.150
P=0.377
P=0.354
P=0.725
B
12 MO
24 MO
eNOS+/-
cc
500 μm
C
eNOS+/+ (12 MO)
eNOS-/- (12 MO)
eNOS+/+ (24 MO)
eNOS-/- (24 MO)
21.5 kDa
18.5 kDa
17 kDa
14 kDa
Degraded?
MBP
β-actin
Relative Protein levels
P=0.0003
P<0.0001
P=0.0077
D
MBP + DAPI
