# Supplementary material for: White matter damage as a consequence of vascular dysfunction in a spontaneous mouse model of chronic mild chronic hypoperfusion with eNOS deficiency
Source: Mol Psychiatry. 2022 Aug 10;27(11):4754–69. doi: 10.1038/s41380-022-01701-9 (PMC9734049; doi:10.1038/s41380-022-01701-9)
Supplement: Supplementary file 2 [file 41380_2022_1701_MOESM2_ESM.pdf]

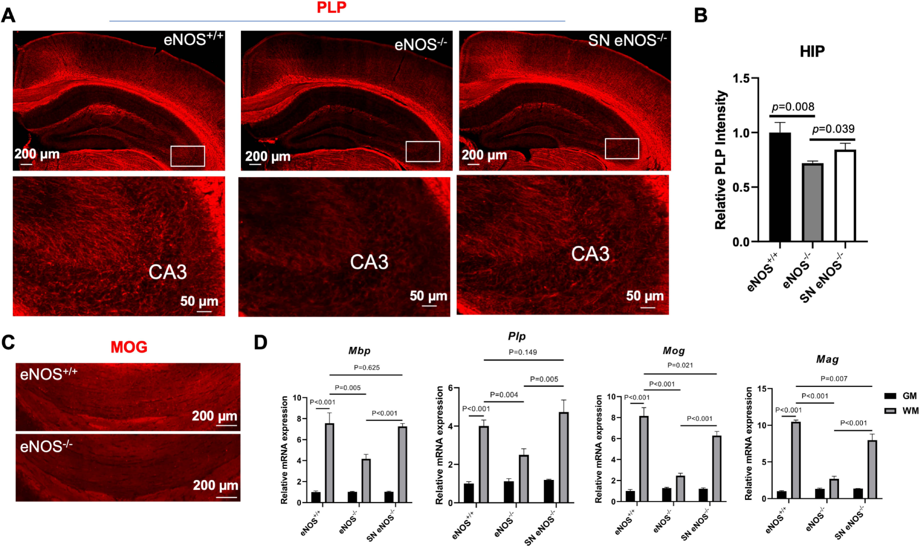
A
PLP
eNOS+/+
eNOS-/-
SN eNOS-/-
200 µm
CA3
50 µm
B
HIP
Relative PLP Intensity
p=0.008
p=0.039
eNOS+/+
eNOS-/-
SN eNOS-/-
C
MOG
eNOS+/+
eNOS-/-
200 µm
D
Mbp
Plp
Mog
Mag
Relative mRNA expression
P<0.001
P=0.005
P=0.625
P=0.004
P=0.149
P=0.021
P=0.007
GM
WM
