# Supplementary material for: White matter damage as a consequence of vascular dysfunction in a spontaneous mouse model of chronic mild chronic hypoperfusion with eNOS deficiency
Source: Mol Psychiatry. 2022 Aug 10;27(11):4754–69. doi: 10.1038/s41380-022-01701-9 (PMC9734049; doi:10.1038/s41380-022-01701-9)
Supplement: Supplementary file 3 [file 41380_2022_1701_MOESM3_ESM.pdf]

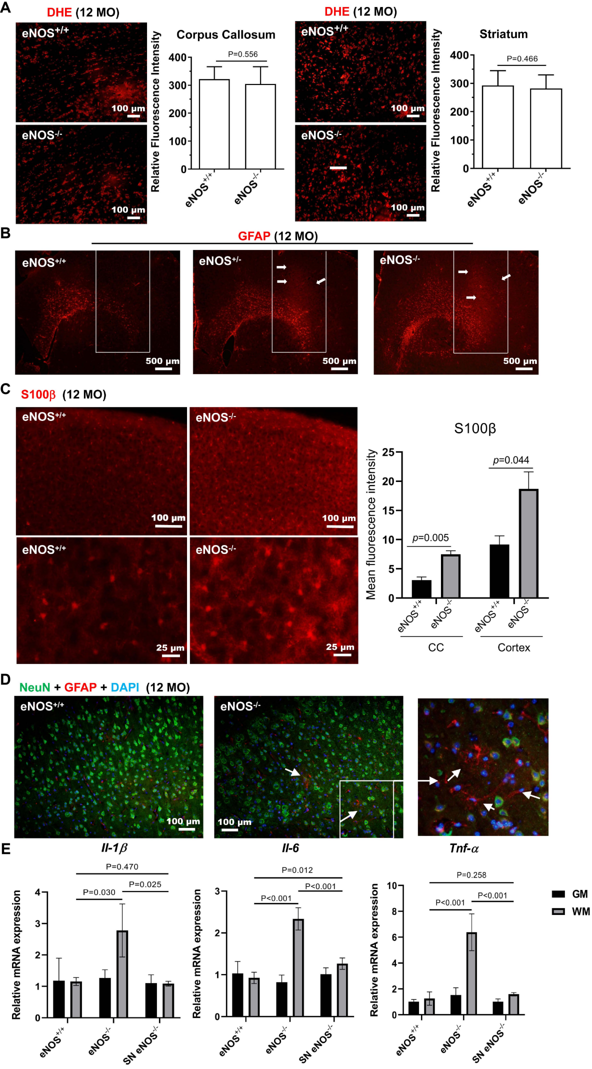
A
DHE (12 MO)
eNOS+/+
eNOS-/-
100 µm
Corpus Callosum
P=0.556
Relative Fluorescence Intensity
400
300
200
100
0
eNOS+/+
eNOS-/-
Striatum
P=0.466
B
GFAP (12 MO)
eNOS+/+
eNOS+/-
eNOS-/-
500 µm
C
S100β (12 MO)
100 µm
25 µm
S100β
Mean fluorescence intensity
25
20
15
10
5
0
p=0.005
p=0.044
CC
Cortex
D
NeuN + GFAP + DAPI (12 MO)
100 µm
E
Il-1β
Il-6
Tnf-α
Relative mRNA expression
P=0.470
P=0.030
P=0.025
P=0.012
P<0.001
P<0.001
P=0.258
P<0.001
P<0.001
SN eNOS-/-
GM
WM
4
3
2
1
0
10
8
6
4
2
0
