# Supplementary material for: White matter damage as a consequence of vascular dysfunction in a spontaneous mouse model of chronic mild chronic hypoperfusion with eNOS deficiency
Source: Mol Psychiatry. 2022 Aug 10;27(11):4754–69. doi: 10.1038/s41380-022-01701-9 (PMC9734049; doi:10.1038/s41380-022-01701-9)
Supplement: Supplementary file 4 [file 41380_2022_1701_MOESM4_ESM.pdf]

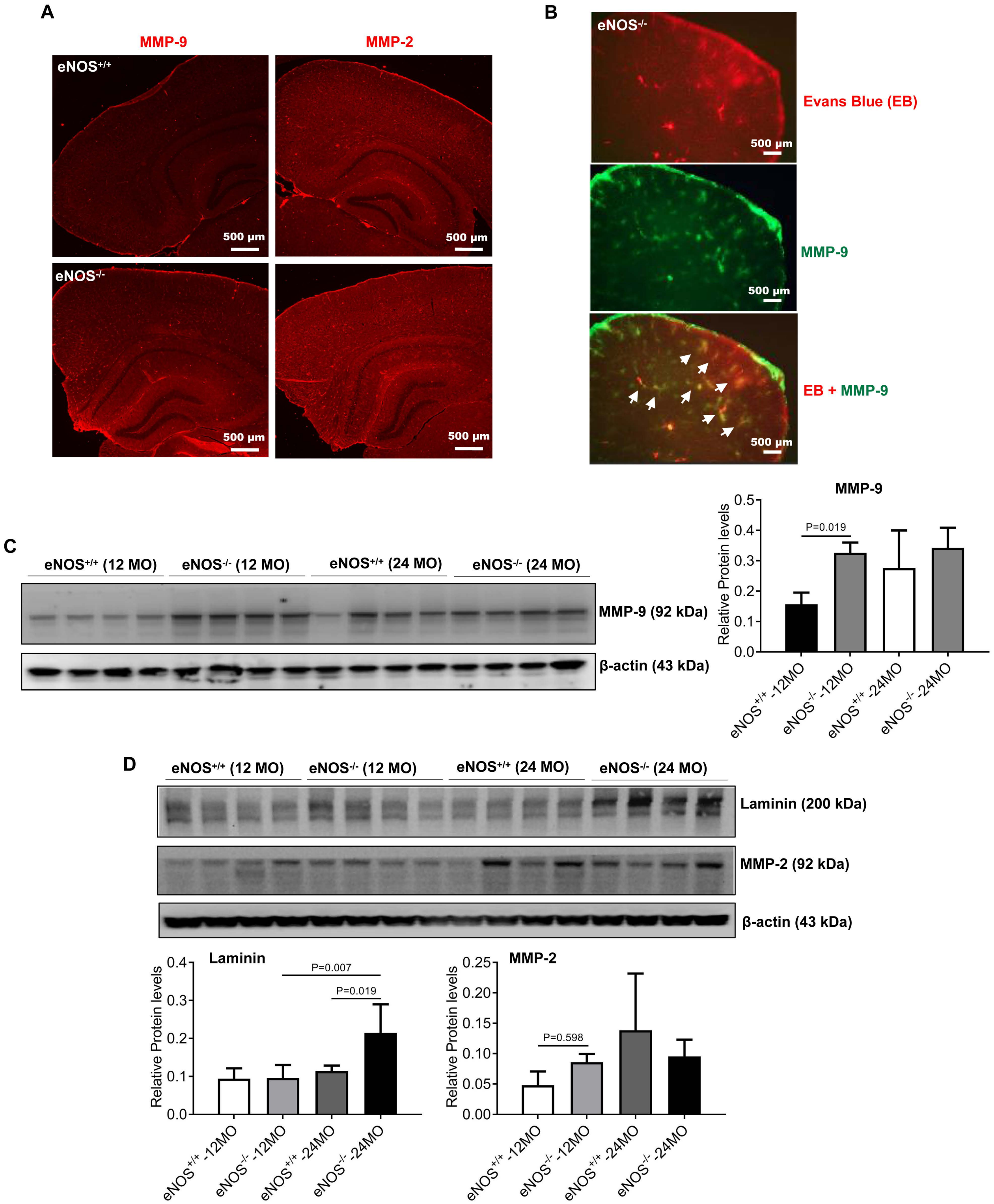

A
MMP-9
MMP-2
eNOS+/+
eNOS-/-
500 µm
B
eNOS-/-
Evans Blue (EB)
MMP-9
EB + MMP-9
500 µm
C
eNOS+/+ (12 MO)
eNOS-/- (12 MO)
eNOS+/+ (24 MO)
eNOS-/- (24 MO)
MMP-9 (92 kDa)
β-actin (43 kDa)
MMP-9
Relative Protein levels
P=0.019
eNOS+/+ -12MO
eNOS-/- -12MO
eNOS+/+ -24MO
eNOS-/- -24MO
D
eNOS+/+ (12 MO)
eNOS-/- (12 MO)
eNOS+/+ (24 MO)
eNOS-/- (24 MO)
Laminin (200 kDa)
MMP-2 (92 kDa)
β-actin (43 kDa)
Laminin
Relative Protein levels
P=0.007
P=0.019
MMP-2
P=0.598
eNOS+/+ -12MO
eNOS-/- -12MO
eNOS+/+ -24MO
eNOS-/- -24MO
