# Supplementary material for: White matter damage as a consequence of vascular dysfunction in a spontaneous mouse model of chronic mild chronic hypoperfusion with eNOS deficiency
Source: Mol Psychiatry. 2022 Aug 10;27(11):4754–69. doi: 10.1038/s41380-022-01701-9 (PMC9734049; doi:10.1038/s41380-022-01701-9)
Supplement: Supplementary file 5 [file 41380_2022_1701_MOESM5_ESM.pdf]

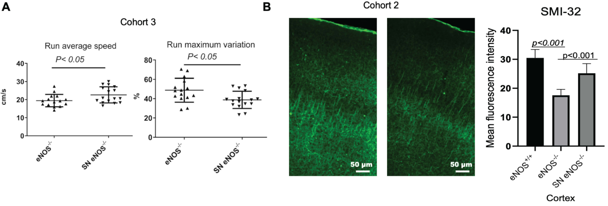
A
Cohort 3
Run average speed
P< 0.05
cm/s
40
30
20
10
0
eNOS-/-
SN eNOS-/-
Run maximum variation
P< 0.05
%
80
60
40
20
0
eNOS-/-
SN eNOS-/-
B
Cohort 2
50 µm
50 µm
SMI-32
Mean fluorescence intensity
40
30
20
10
0
p<0.001
p<0.001
eNOS+/+
eNOS-/-
SN eNOS-/-
Cortex
