# Supplementary material for: White matter damage as a consequence of vascular dysfunction in a spontaneous mouse model of chronic mild chronic hypoperfusion with eNOS deficiency
Source: Mol Psychiatry. 2022 Aug 10;27(11):4754–69. doi: 10.1038/s41380-022-01701-9 (PMC9734049; doi:10.1038/s41380-022-01701-9)
Supplement: Supplementary file 6 [file 41380_2022_1701_MOESM6_ESM.pdf]

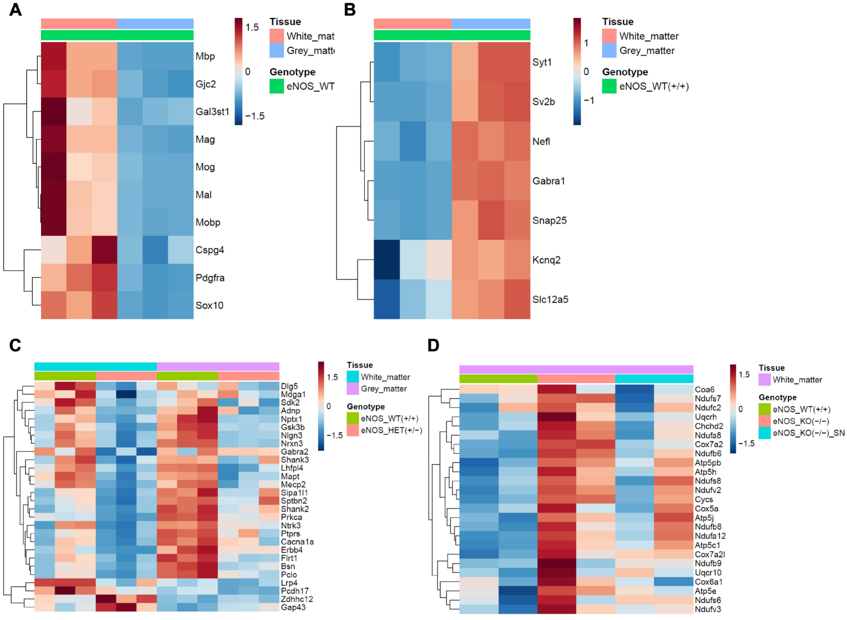
A
Tissue
White_mat
Grey_matt
Genotype
eNOS_WT
1.5
0
−1.5
Mbp
Gjc2
Gal3st1
Mag
Mog
Mal
Mobp
Cspg4
Pdgfra
Sox10
B
Tissue
White_matter
Grey_matter
Genotype
eNOS_WT(+/+)
1
0
−1
Syt1
Sv2b
Nefl
Gabra1
Snap25
Kcnq2
Slc12a5
C
Tissue
White_matter
Grey_matter
Genotype
eNOS_WT(+/+)
eNOS_HET(+/−)
1.5
0
−1.5
Dlg5
Mdga1
Sdk2
Adnp
Nptx1
Gsk3b
Nlgn3
Nrxn3
Gabra2
Shank3
Lhfpl4
Mapt
Mecp2
Sipa1l1
Sptbn2
Shank2
Prkca
Ntrk3
Ptprs
Cacna1a
Erbb4
Flrt1
Bsn
Pclo
Lrp4
Pcdh17
Zdhhc12
Gap43
D
Tissue
White_matter
Genotype
eNOS_WT(+/+)
eNOS_KO(−/−)
eNOS_KO(−/−)_SN
1.5
0
−1.5
Coa6
Ndufs7
Ndufc2
Uqcrh
Chchd2
Ndufa8
Cox7a2
Ndufb6
Atp5pb
Atp5h
Ndufs8
Ndufv2
Cycs
Cox5a
Atp5j
Ndufb8
Ndufa12
Atp5c1
Cox7a2l
Ndufb9
Uqcr10
Cox6a1
Atp5e
Ndufs6
Ndufv3
