# Supplementary material for: White matter damage as a consequence of vascular dysfunction in a spontaneous mouse model of chronic mild chronic hypoperfusion with eNOS deficiency
Source: Mol Psychiatry. 2022 Aug 10;27(11):4754–69. doi: 10.1038/s41380-022-01701-9 (PMC9734049; doi:10.1038/s41380-022-01701-9)
Supplement: Supplementary file 7 [file 41380_2022_1701_MOESM7_ESM.pdf]

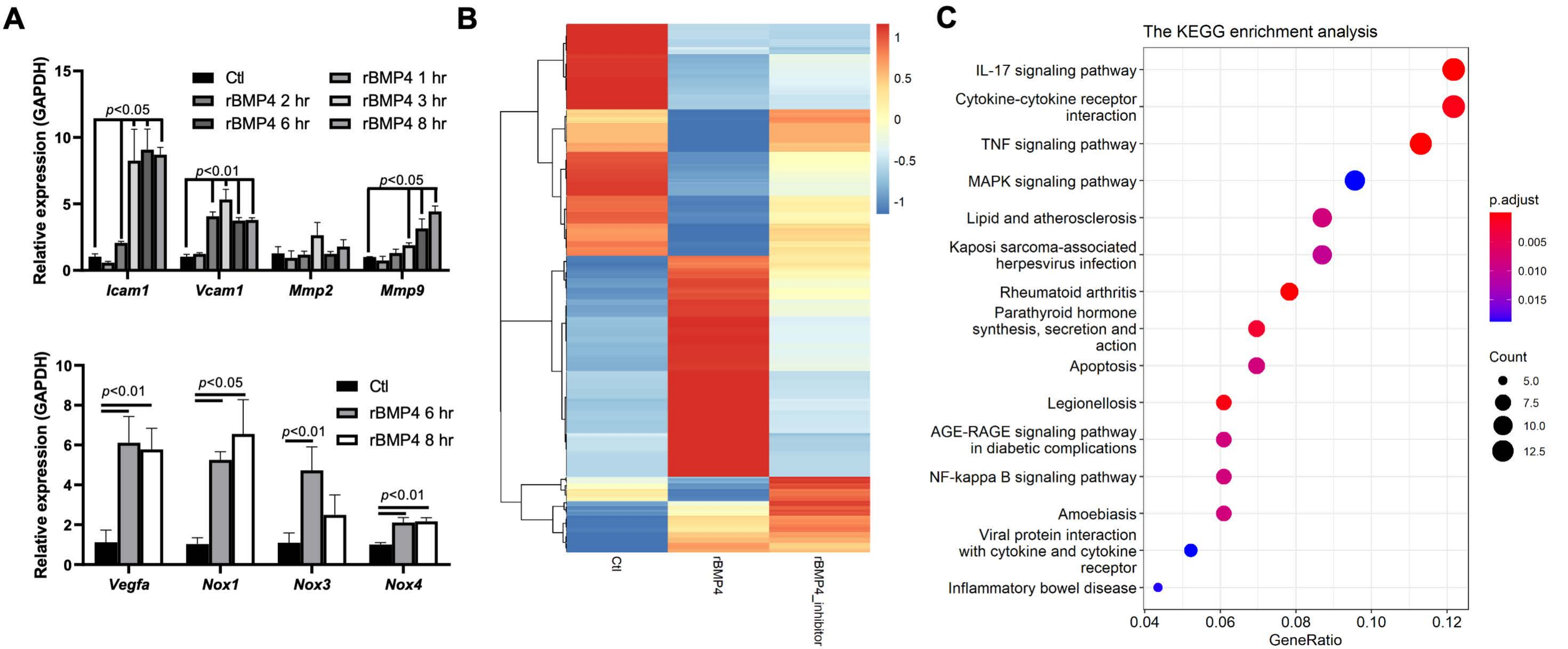
A
Relative expression (GAPDH)
15
10
5
0
Ctl
rBMP4 1 hr
rBMP4 2 hr
rBMP4 3 hr
rBMP4 6 hr
rBMP4 8 hr
p<0.05
p<0.01
p<0.05
Icam1
Vcam1
Mmp2
Mmp9
Relative expression (GAPDH)
10
8
6
4
2
0
p<0.01
p<0.05
p<0.01
p<0.01
Ctl
rBMP4 6 hr
rBMP4 8 hr
Vegfa
Nox1
Nox3
Nox4
B
1
0.5
0
-0.5
-1
Ctl
rBMP4
rBMP4_inhibitor
C
The KEGG enrichment analysis
IL-17 signaling pathway
Cytokine-cytokine receptor interaction
TNF signaling pathway
MAPK signaling pathway
Lipid and atherosclerosis
Kaposi sarcoma-associated herpesvirus infection
Rheumatoid arthritis
Parathyroid hormone synthesis, secretion and action
Apoptosis
Legionellosis
AGE-RAGE signaling pathway in diabetic complications
NF-kappa B signaling pathway
Amoebiasis
Viral protein interaction with cytokine and cytokine receptor
Inflammatory bowel disease
0.04
0.06
0.08
0.10
0.12
GeneRatio
p.adjust
0.005
0.010
0.015
Count
5.0
7.5
10.0
12.5
