# Supplementary material for: White matter damage as a consequence of vascular dysfunction in a spontaneous mouse model of chronic mild chronic hypoperfusion with eNOS deficiency
Source: Mol Psychiatry. 2022 Aug 10;27(11):4754–69. doi: 10.1038/s41380-022-01701-9 (PMC9734049; doi:10.1038/s41380-022-01701-9)
Supplement: Supplementary file 8 [file 41380_2022_1701_MOESM8_ESM.pdf]

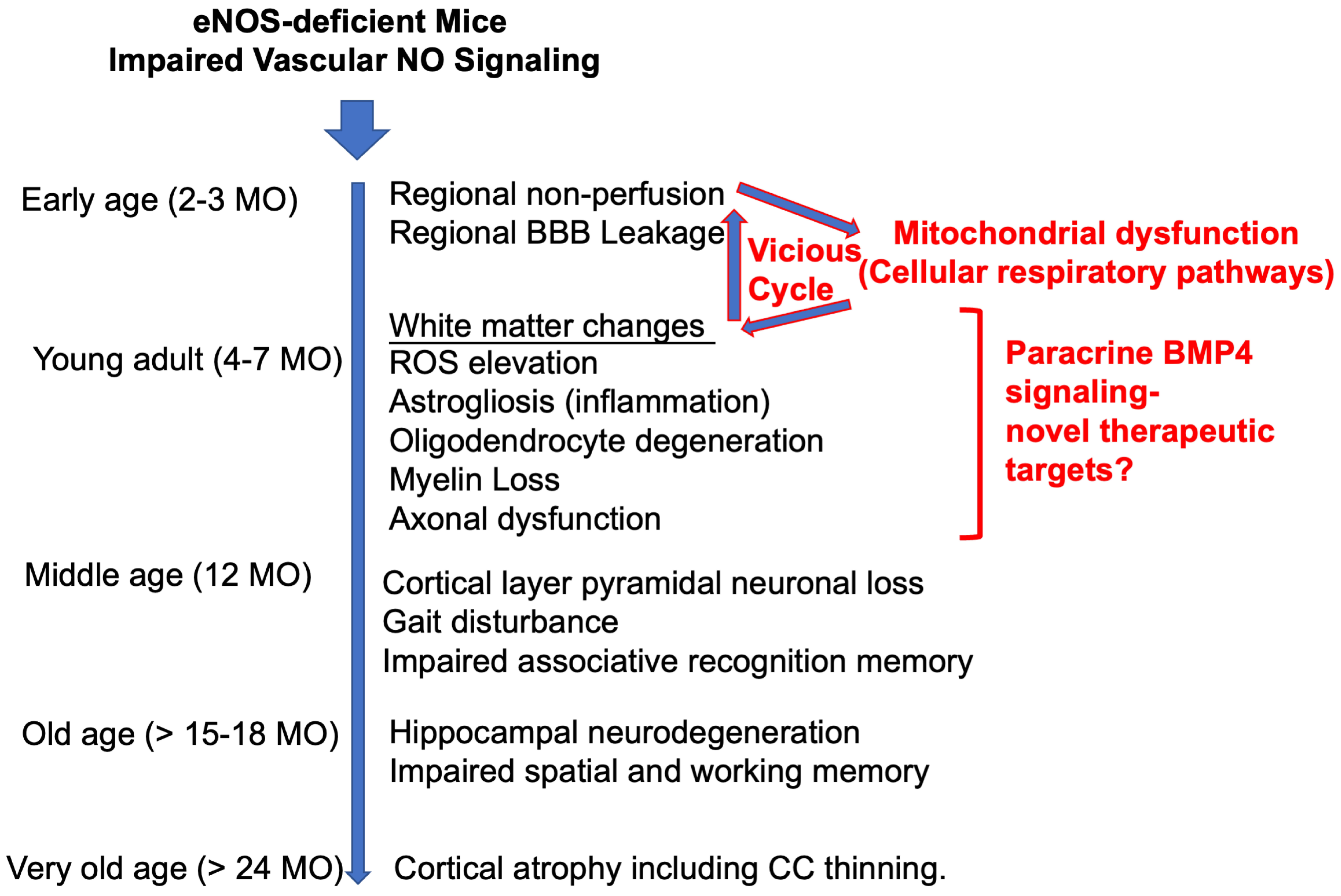
eNOS-deficient Mice
Impaired Vascular NO Signaling
Early age (2-3 MO)
Regional non-perfusion
Regional BBB Leakage
Vicious Cycle
Mitochondrial dysfunction
(Cellular respiratory pathways)
Young adult (4-7 MO)
White matter changes
ROS elevation
Astrogliosis (inflammation)
Oligodendrocyte degeneration
Myelin Loss
Axonal dysfunction
Paracrine BMP4 signaling- novel therapeutic targets?
Middle age (12 MO)
Cortical layer pyramidal neuronal loss
Gait disturbance
Impaired associative recognition memory
Old age (> 15-18 MO)
Hippocampal neurodegeneration
Impaired spatial and working memory
Very old age (> 24 MO)
Cortical atrophy including CC thinning.
